# Novel reassortant swine H3N2 influenza A viruses in Germany

**DOI:** 10.1038/s41598-020-71275-5

**Published:** 2020-08-31

**Authors:** Roland Zell, Marco Groth, Andi Krumbholz, Jeannette Lange, Anja Philipps, Ralf Dürrwald

**Affiliations:** 1 Section of Experimental Virology, Institute for Medical Microbiology, Jena University Hospital, Friedrich Schiller University Jena, 07745 Jena, Germany; 2 CF DNA Sequencing, Leibniz Institute on Aging, Fritz Lipmann Institute, 07745 Jena, Germany; 3 Present Address: Institute of Infection Medicine, Kiel University and University Medical Center Schleswig-Holstein, 24105 Kiel, Germany; 4 Present Address: Paul-Ehrlich-Institut, 63225 Langen, Germany; 5 Present Address: Thermo Fisher Scientific GENEART GmbH, 93059 Regensburg, Germany; 6 Present Address: Robert Koch Institute, 13353 Berlin, Germany

**Keywords:** Diseases, Infectious diseases, Influenza virus

## Abstract

Analysis of 228 H3N2 swine influenza A virus isolates collected between 2003 and 2015 in Germany revealed important changes in molecular epidemiology. The data indicate that a novel reassortant, Rietberg/2014-like swine H3N2, emerged in February 2014 in Northern Germany. It is comprised of a hemagglutinin gene of seasonal H3N2 (A/Denmark/129/2005-like), a neuraminidase gene of Emmelsbuell/2009-like swine H1N2 and the internal gene cassette of pandemic H1N1 viruses. Together with Danish swine H3N2 strains of 2013–2015 with identical genome layout, the Rietberg/2014-like viruses represent a second swine H3N2 lineage which cocirculates with a variant of the Gent/1984-like swine H3N2 lineage. This variant, named Gent1984/Diepholz-like swine H3N2, has a Gent/1984-like HA and a Diepholz/2008-like NA; the origin of the internal gene cassette likely derived from avian-like swine H1N1. The first isolate of the Gent1984/Diepholz reassortant emerged in Northern Germany in September 2011 whereas the last German Gent/1984-like isolate was collected in October 2011.

## Introduction

Influenza of pigs caused by swine influenza A viruses (IAV-S) is a condition that effects considerable economic losses. Diseased pigs suffer from fever and acute bronchitis associated with laboured breathing, lethargy and weight loss. One sequela of swine influenza is reproductive failure of sows. Usually, the disease is benign and the pigs recover after 2–6 days. Measures to control swine influenza include changes in herd management and vaccination. The prevalence of swine influenza varies in European countries as shown by the recent ESNIP3 survey that investigated more than 9,000 herds in 17 countries^1^. On average, IAV-S was detected in 31% of the herds. A subsequent analysis of 290 IAV-S isolates collected from 14 European countries revealed a remarkable genetic diversity^2^. This is the result of (i) reassortment events upon multiple infection, and (ii) ecological factors, e.g. a broad host range of IAV which allows a limited exchange of virus between aquatic fowl (main hosts), poultry, pigs, humans, and horses. Of special relevance are zoonotic infections of humans and anthroponotic (reverse-zoonotic) infections of pigs. Emergence of A(H1N1)pdm2009 in the human population and of Eurasian human-like swine H1N2 (hu-like swH1N2) as well as human-like swine H3N2 (hu-like swH3N2) in pigs are recent examples (reviewed in Ref.^3^). Genetic drift, adaptation to specific hosts and geographic isolation are the main drivers that led to the evolution of sublineages and alleles of all IAV gene segments, which have been described in a previous study by Lu et al.^4^.

We have recently reported on a large-scale investigation of swH1N2 IAV-S conducted by members of the German FluResearchNet consortium^5^. Genome sequencing and genetic analysis of 267 swH1N2 isolates collected during a 13-year long-term swine influenza surveillance in Germany revealed the replacement of the previously prevalent European continental hu-like swH1N2 by four novel swH1N2 reassortants named Diepholz/2008-like, Emmelsbuell/2009-like, Papenburg/2010-like and Gladau/2012-like swH1N2. Here, we describe the genetic analysis of 228 swH3N2 isolates collected in the same survey. The data indicate that a triple reassortant swH3N2 emerged with a seasonal A/Denmark/129/2005-like hemagglutinin (HA) gene, the neuraminidase (NA) gene of an Emmelsbuell/2009-like swH1N2 virus and the internal gene cassette (IGC) of A(H1N1)pdm2009 (pdm09 IGC). Secondly, the continental hu-like swH3N2 viruses (Gent/1984-like) were replaced by a variant swH3N2 with a similar gene constellation in Germany.

## Results

### Study design and nomenclature of IAV-S lineages and clades

**Figure 1:**
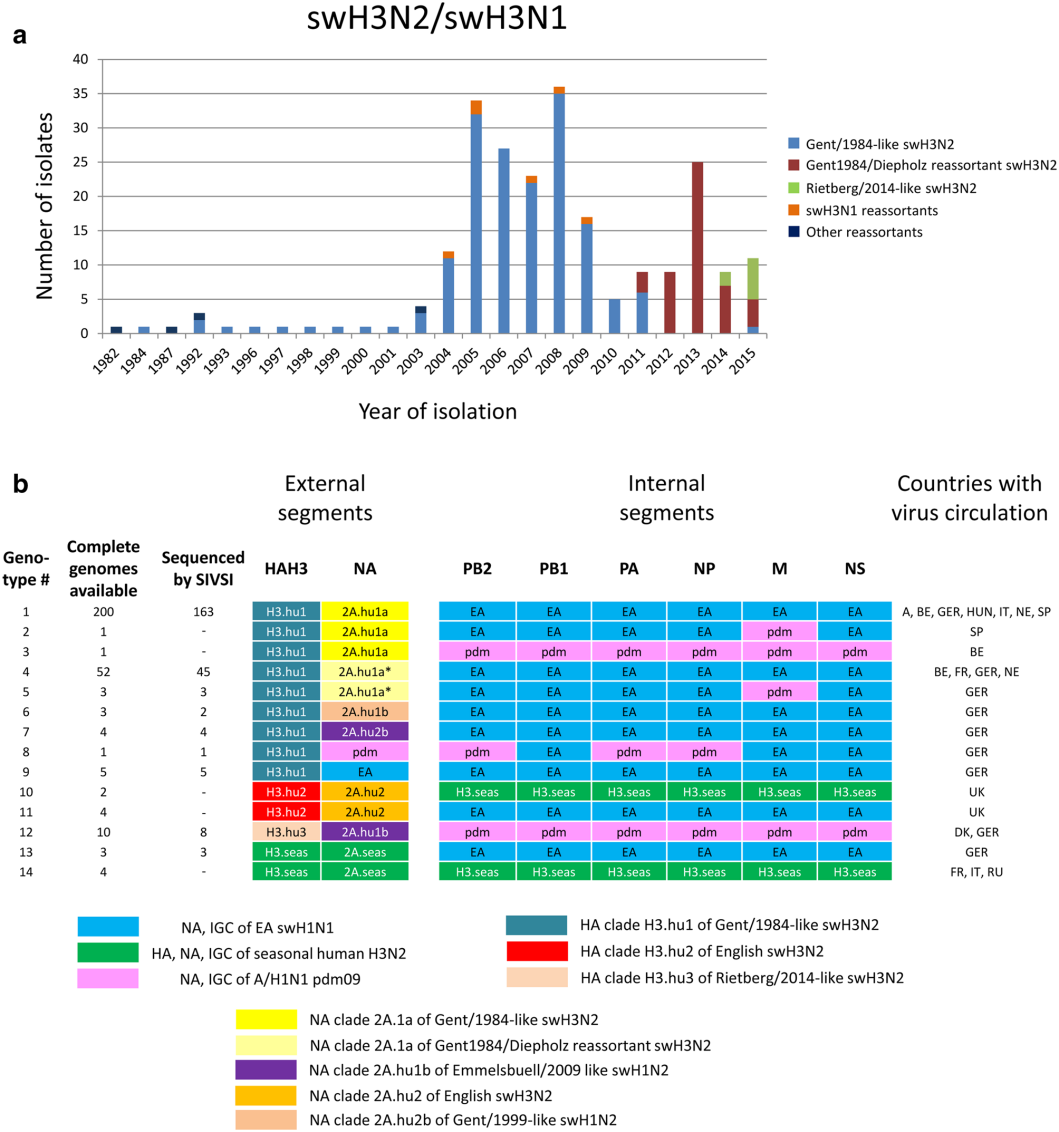
Genetically characterized swH3N2 and swH3N1 isolates from Europe. (**a**) Numbers of isolates sequenced in this study plotted against the year of isolation and itemized by clades. (**b**) Genotypes of complete swH3N2 and swH3N1 genomes sequenced by the Jena Swine Influenza Virus Sequencing Initiative (SIVSI) consortium or available from the GenBank and EpiFlu databases. Abbreviations: H3.seas, gene segments of the seasonal human H3N2; H3.hu1, H3.hu2, H3.hu3, HA sublineages of IAV-S derived from the human seasonal HAH3 gene; 2A.hu1a, 2A.hu1b, 2A.hu2, 2A.hu2b, NA sublineages derived from the human seasonal NAN2 gene (2A.seas); 2A.hu1a*, drift variant of the 2A.hu1a sublineage (derived from Diepholz/2008-like swH1N2); EA, gene segments derived from the European avian-like swH1N1; pdm, gene segments derived from A(H1N1)pdm09; abbreviations of countries: *A* Austria, *BE* Belgium, *DK* Denmark, *FR* France, *GER* Germany, *HUN* Hungary, *IT* Italy, *NE* the Netherlands, *SP* Spain, *UK* United Kingdom.

Between February 2003 and December 2015 members of the German FluResearchNet consortium conducted a long-term swine influenza surveillance and tested specimens of 8,122 sampling events (96% swabs, 2% bronchoalveolar lavages, 2% lung tissue samples) for the presence of IAV-S^5^. The specimens were sent in by veterinarians from 12 of 16 German States; few samples were from Austria, Belgium, Denmark, the Netherlands, Spain and Switzerland. The IAV-S positivity rate in PCR was 30.5% and 1,310 IAV-S isolates were gained after inoculation of embryonated hens' eggs. In parallel, 928 strains were isolated in Madin-Darby bovine kidney (MDBK) cells. In addition, our IAV-S archive included 13 swH3N2 isolates that were obtained from other sources (collection dates 1982–2001). Of this collection, 228 swH3N2 plus six swH3N1 isolates were sequenced by the *Jena Swine Influenza Virus Sequencing Initiative* (SIVSI) that was continuously run from 2006 through 2016 (Fig. 1a).

Designations of IAV-S lineages like Eurasian avian (EA) swH1N1, hu-like swH1N2 and hu-like swH3N2 are established in literature and were retained. For subgrouping of the HAH3 gene, the swH3N2 clades were designated H3.hu1, H3.hu2 and H3.hu3 to indicate the human seasonal origin of the HA gene and to distinguish these sublineages from avian and equine lineages. Reassortant Diepholz/2008-like, Emmelsbuell/2009-like, Papenburg/2010-like, Gladau/2012-like swH1N2 viruses and sublineages of the NAN2 gene (denoted 2A.hu1a, 2A.hu1b, 2A.hu2, 2A.hu2a, 2A.hu2b, and 2A.seas) have been introduced recently^5^. The novel reassortant viruses described here were named Rietberg/2014-like swH3N2 and Gent1984/Diepholz-like swH3N2. The prefix sw (swine) indicates the porcine host. Seasonal human H3N2 and A(H1N1)pdm2009 virus were distinguished from porcine strains by adding the subscripts 'seas' and 'pdm' to type designations (H3_seas_N2_seas_, H1_pdm_N1_pdm_).

Figure 1 shows the annual distribution of sequenced isolates and their genome layouts. Altogether 14 genotypes have been detected in European pigs since 1977. The Gent/1984-like swH3N2 (Fig. 1b, genotype #1) have been dominant and were isolated between March 2003, when the survey started, and October 2011 when the last strain of this lineage was collected in Germany. Additional isolates of 1993–2001 indicate their circulation even in the 1990s in Germany. In September 2011, a Gent1984/Diepholz reassortant swH3N2 (#4) emerged at the German/Dutch border and replaced the Gent/1984-like viruses. Another swH3N2 reassortant (#12) came up in 2014, the Rietberg/2014-like swH3N2. In addition, further German H3N2 reassortants with HA and NA genes of seasonal human H3N2 and the IGC of EA swH1N1 (EA IGC) were isolated in 1982, 1984 and 1992 (#13) as well as six H3N1 reassortants of 2004–2009 (#8, #9).

### Analysis of the HA gene

**Figure 2:**
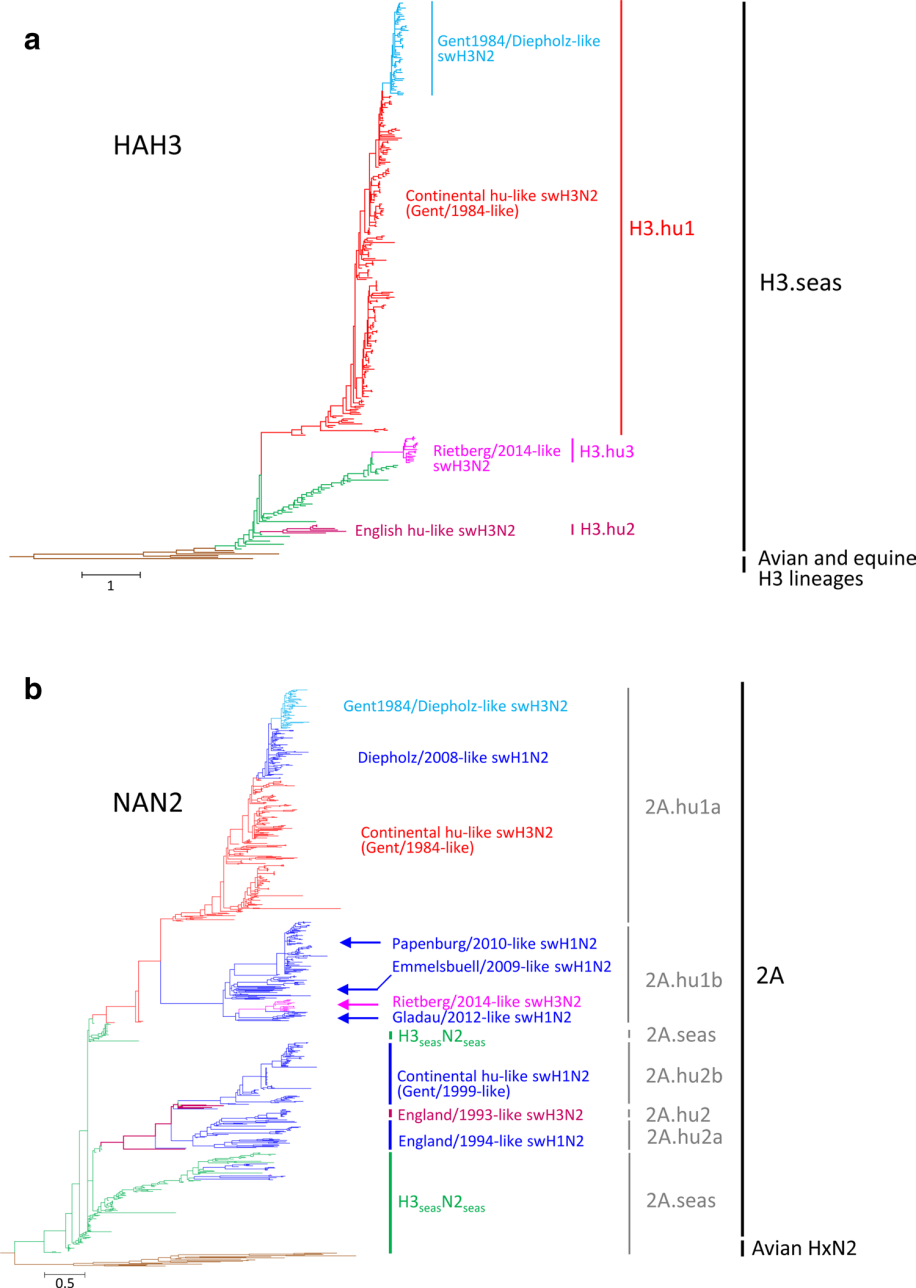
Phylogenetic analyses of HA and NA genes using Bayesian tree inference. Relevant lineages and sublineages are indicated. The scale bars indicate substitutions per site. (**a**) Analysis of 347 HAH3 sequences of the seasonal H3 lineage. Details of strains included in this analysis are presented in Supplementary Fig. 1. Colour code: green, H3_seas_N2_seas_ sequences; brown, avian and equine reference sequences; red, swH3N2 lineage H3.hu1 sequences; purple, swH3N2 lineage H3.hu2 sequences; magenta, swH3N2 lineage H3.hu3 sequences; light blue, Gent1984/Diepholz-like swH3N2. (**b**) Analysis of 841 NAN2 sequences. Details of strains included in this analysis are presented in Supplementary Fig. 3. Colour code: red, swH3N2 strains with H3.hu1 HA; purple, swH3N2 strains wih H3.hu2 HA; magenta, swH3N2 strains with H3.hu3 HA; dark blue, swH1N2 sequences; light blue, Gent1984/Diepholz-like swH3N2; green: human seasonal H3N2 sequences, brown: avian NAN2 sequences (for reference).

**Figure 3:**
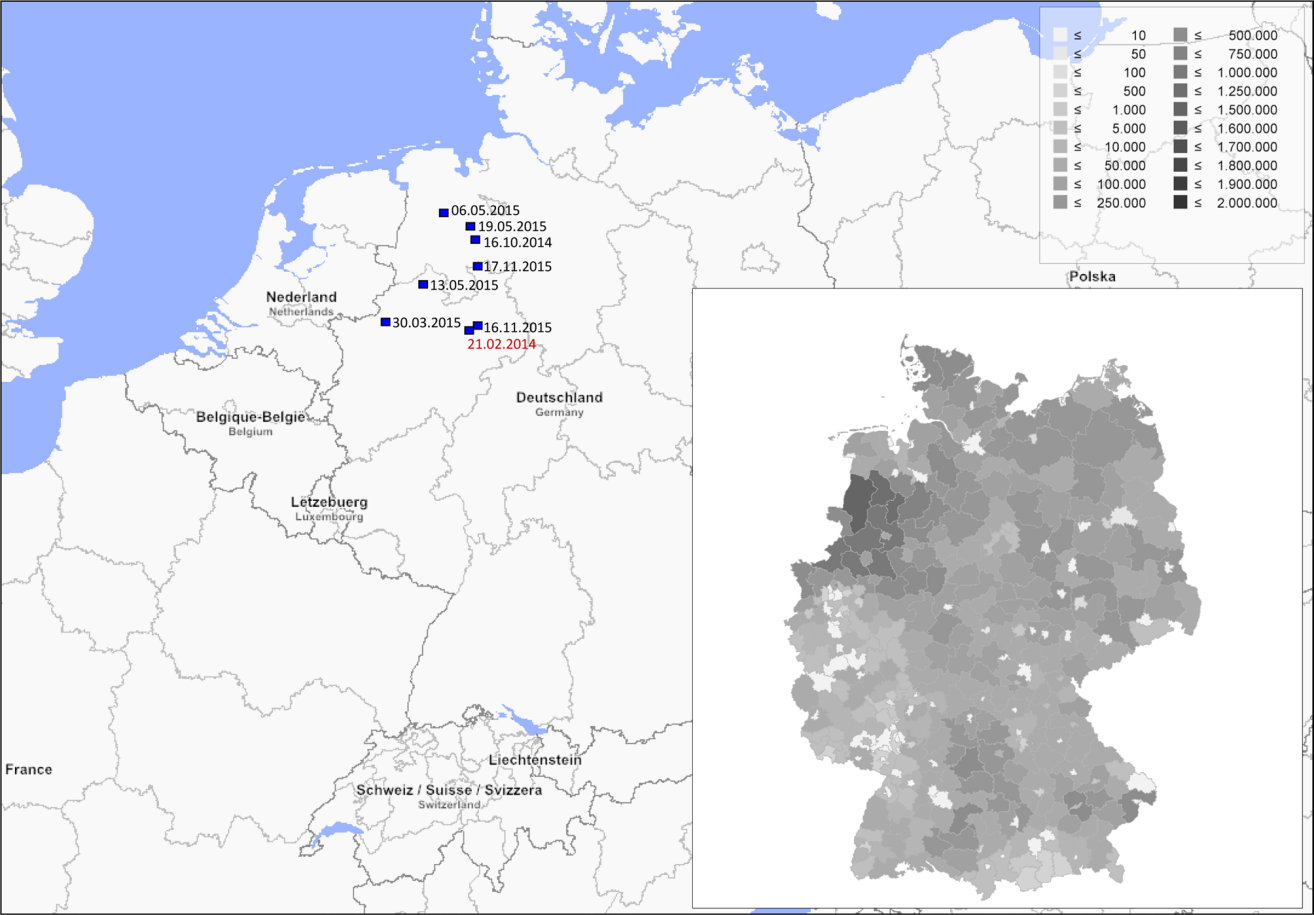
Incursion of Rietberg/2014-like swH3N2 into the German pig population. Dark squares indicate the places where virus-positive swabs were collected; collection dates (dd.mm.yyyy) are given; the sampling date of the first German isolate is printed in red. The inset presents the pig population density in Germany (numbers of pigs per administrative district). The data were retrieved from the 2017-yearbooks of the 16 State Statistical Offices in Germany and visualized with Regiograph Analyse software, version 2019 (distributed by GfK Geomarketing GmbH, Germany, download at https://regiograph.gfk.com).

For phylogenetic analysis of the HA segment, we compiled 300 H3 sequences of IAV-S and 47 H3 sequences of other hosts for reference (one equine, five avian, 41 human strains). Altogether 347 sequences were included, 234 of which were IAV-S sequences that had been generated by SIVSI (78.0% of IAV-Ss). The results are presented in Fig. 2 and Supplementary Fig. 1. The data reveal three distinct HA sequence clusters of European hu-like swH3N2 in the human seasonal H3 lineage, denoted in chronological order H3.hu1, H3.hu2 and H3.hu3 (Fig. 2a). Cluster H3.hu1 includes the HA sequences of the continental hu-like swH3N2 (Gent/1984-like swH3N2) (Fig. 1b, genotype #1 and derivatives: #2, #3, #6, #7, #8, #9). These viruses showed a wide dispersal and have been prevalent in Germany, 1993–2011. The tip of the H3.hu1 cluster is comprised of a clade of 55 sequences of the Gent1984/Diepholz reassortant (genotype #4 and three derivative strains: #5). Whereas the HA protein of these viruses is inconspicious (only one amino acid substitution, V546I, is characteristic of this group), the NA clearly derived from the Diepholz/2008-like swH1N2 (see below). Cluster H3.hu2 includes the HA sequences of anthroponotic H3_seas_N2_seas_ strains (two isolates of 1987 and 1990, #10) and of the England/375017/1993-like swH3N2 that circulated in the UK only, 1993–1997 (#11). The latter viruses are 6 + 2 reassortants of Eurasian avian (EA) swH1N1 (donor of EA IGC) and H3_seas_N2_seas_ (donor of HA and NA, #10). The England/375017/1993-like swH3N2 came up soon after emergence of the EA swH1N1 in the UK, 1992. Cluster H3.hu3 includes the HA sequences of a triple reassortant with an unusual gene constellation. This cluster is represented by isolate A/swine/Rietberg/19732/2014 (genotype #12) and comprises eight German and nine Danish strains. Rietberg/2014-like swH3N2 emerged in northwest Germany in February 2014 and spread to several swine holdings with up to 100 km distance (Fig. 3). Northwest Germany has the highest pig population density in Germany (compare inlet of Fig. 3). The HA tree shows the close phylogenetic relationship (i) of Gent/1984-like swH3N2 with A/Port Chalmers/1/1973, (ii) of England/1993-like swH3N2 with A/Albany/20/1974, and (iii) of the Rietberg/2014-like swH3N2 with more recent seasonal H3N2 viruses (Fig. 2, Supplementary Fig. 1). A detailed phylogenetic analysis of the origin of the Rietberg/2014-like HA gene was conducted including 98 seasonal H3N2 (H3_seas_N2_seas_) strains collected between 1968 and 2013 (Supplementary Fig. 2, left panel). This HA tree revealed A/Denmark/129/2005 as closest known relative. Other related H3_seas_N2_seas_ viruses like A/Bayern/4/2006, A/Berlin/2/2006 and A/Lyon/636/2006 were excluded in our analysis due to the availability of partial sequence data only.

### Analysis of the NA gene

Our previous study investigated the evolution of hu-like swH1N2 strains in Germany, 2000–2015^5^, including the phylogenetic analysis of all gene segments. Here, we extended the previous NA analysis and investigated an alignment of 841 NA sequences comprising 668 IAV-S (293 swH3N2, 375 swH1N2) plus 153 H3_seas_N2_seas_ strains and 20 avian strains for reference. Altogether 491 of the IAV-S strains of this alignment (73.5%) have been sequenced by SIVSI.

The general topology of the N2 tree (Fig. 2b, Supplementary Fig. 3) is complex and comprises avian N2 lineages, the human seasonal N2 lineage (2A.seas) and five IAV-S clades designated (i) sublineage 2A.hu1a (the Gent/1984-like swH3N2), (ii) sublineage 2A.hu1b (derivative IAV-S strains with NA of the Gent/1984-like viruses and various HA genes), and (iii) sublineage 2A.hu2 (the English swH3N2), (iv) sublineage 2A.hu2a (the English swH1N2), and (v) sublineage 2A.hu2b (the Gent/1999-like swH1N2). The Rietberg/2014-like swH3N2 cluster together with the Emmelsbuell/2009-like, Papenburg/2010-like and Gladau/2014-like swH1N2 in one branch suggesting that a single, initial reassortment event led to the evolution of the NA 2A.hu1b sublineage. We identified three swH3N2 strains with apparent relationship to the unknown NA donor. These are A/swine/Denmark/14348-9/2003, A/swine/Muesleringen-S./IDT4317/2005 and A/swine/Hertzen/IDT4317/2005. Notably, these three strains exhibit numerous amino acid substitutions in the NA and HA proteins and cluster close the roots of the HA H3.hu1 and NA 2A.hu1b branches.

At the tip of the 2A.hu1a branch is a cluster of swH1N2 strains which has been described previously as the Diepholz/2008-like reassortant^5^. Interestingly, this reassortant gave rise to a monophyletic cluster of more than 50 swH3N2 strains isolated from 2011 onwards. This NA tree tropology is compatible with the assumption of two consecutive reassortment events. The first reassortment led to the emergence of the Diepholz/2008-like swH1N2, the second reassortment included Gent/1984-like swH3N2 (HA donor) and Diepholz/2008-like swH1N2 (NA donor) and resulted in a Gent1984/Diepholz reassortant with identical HA/NA features compared to the HA donor (i.e., H3.hu1 and 2A.hu1a) but a different IGC configuration (see below). Analysis of the collection dates revealed that no Gent/1984-like swH3N2 have been collected in Germany soon after emergence of the Gent1984/Diepholz reassortant swH3N2 in 2011; the former, however, still circulated in Belgium (2012), Italy (2013), Hungary (2013) and Spain (2015).

### Analysis of the internal gene cassette (IGC)

Phylogenetic analyses of the IGC gene segments have been conducted to further investigate the novel reassortants. The trees demonstrate that Gent1984/Diepholz reassortant viruses have an EA IGC most likely derived from an EA swH1N1 donor suggesting a triple reassortant (Supplementary Fig. 4A–F). This hypothesis is supported by the following findings: (i) The Gent1984/Diepholz reassortant has a full-length NS1 protein whereas the Gent/1084-like swH3N2 and the Diepholz/2008-like swH1N2 have not (Supplementary Fig. 4F). (ii) Lack of clustering excludes a direct ancestry of the Gent/1984-like swH3N2 or the Diepholz/2008-like swH1N2 whereas EA swH1N1 strains are the closest relatives in all trees. Hence, Gent1984/Diepholz reassortant swH3N2 is likely triple reassortant. In contrast, the Rietberg/2014-like viruses have a pdm09 IGC with truncated versions of PB1-F2, PA-X and NS1.

### Antigenic characterization

The evolution of Gent/1984-like swH3N2 was accompanied by a moderate antigenic drift over the years which covers a decrease of 2 log_2_ of hemagglutination inhibition titers in average, 2003–2011 (Table 1). The Gent1984/Diepholz reassortant is comprised of viruses with remarkably lower cross-reactivity to Gent/1984-like swH3N2 by 3–4 log_2_ titers. For investigation of the antigenic sites, the respective HA sequences of Gent1984/Diepholz-like strains were compared to the other sequences of the HA alignment. The following amino acids as defined by Wiley et al., Smith et al. and Nakajima et al. were considered: site A (aa 122, 124, 131, 133, 135, 137, 138, 140–146), B (aa 155–160, 163, 164, 187–197), C (aa 53, 54, 275–278), D (aa 172–174, 201, 207, 213, 217, 225–230, 242–244), and E (aa 62, 63, 78, 82, 83, 260–262)^6–8^. Six substitutions were observed in the antigenic sites of A/swine/Guenne/21206/2015 (site A: P143L, N145K; site B: L164Q, R189K, N193D; site D: F174Y) and three in A/swine/Telgte/19839/2014 (site B: L164Q, R189I, N193K). This indicates a significant antigenic drift of site B of the more recent Gent1984/Diepholz-like strains (Supplementary Fig. 5).

**Table 1:** Geometric mean of hemagglutination inhibiting units measured by testing immune sera of 10 pigs immunized with A/swine/Bakum/IDT1769/2003 (H3N2) against the corresponding virus.

Gent/1984-like swH3N2			Gent1984/Diepholz reassortant swH3N2		
Bakum/IDT1769/2003	Coburg/11210/2009	Visbek/13347/2011	Haren/13906/2011	Telgte/19839/2014	Günne/21206/2015
520	321	140	98	27	49

Heamagglutination inhibiting unit, hemagglutination inhibition titre reciprocal.

The hemagglutinin of the viruses of the Rietberg/2014-sublineage did not show cross-reactivity to the Gent/1984-like viruses but reacted well with a seasonal influenza virus of 2007 (Table 2).

**Table 2:** Antigenic profiles of the Gent/1984-like and Rietberg/2014-like swH3N2 viruses in comparison to mid-2000s seasonal H3N2 of humans.

Antiserum	Virus							
	A/swine/Gent/220/1992 (Gent/1984-like swH3N2)		A/swine/Bakum/IDT1769/2003 (Gent/1984-like swH3N2)		A/swine/Rietberg/19732/2014 (H3N2)		A/Brisbane/10/2007 (H3N2)	
	HI^a^	NT^a^	HI	NT	HI	NT	HI	NT
A/swine/Gent/220/1992	20,480	82,783	20,480	20,321	< 20	< 13	< 20	< 13
A/swine/Bakum/IDT1769/2003	320	3,221	640	1,282	< 20	< 13	< 20	< 13
A/swine/Rietberg/19732/2014	< 20	< 13	< 20	< 13	2,560	5,223	1,280	3,221
A/Brisbane/10/2007	< 20	< 13	< 20	< 13	1,280	83,176	5,120	131,202

Determined by using hyper immune sera.

^a^Reciprocal titers are given.

The antigenic sites of the HA sequences of Rietberg/2014-like strains were compared to the sequences of both HA alignments (Supplementary Figs. 2, 5). There are no publicly available German full-length HA sequences of H3_seas_N2_seas_ strains of that time which match the antigenic properties of the Rietberg/2014-like viruses. Hence, we used the closely related A/Denmark/129/2005 as a reference strain. This virus belongs to a phylogenetic subgroup which was characterized by G50R, V112I, K173E and N145S exchanges (other representative strains: A/Bayern/4/2006, A/Berlin/2/2006, A/Lyon/636/2006 according to the *September 2006 interim report* of the Worldwide Influenza Centre; https://www.crick.ac.uk/partnerships/worldwide-influenza-centre/annual-and-interim-reports, accessed 12 September 2019). All but one of these exchanges (K173E) are present in Rietberg/2014-like strains. Instead of K173E we observed K173D which is one of eight substitutions of antigenic sites A (D_133_, K_135_, S_138_, K_144_), B (G_158_, K_197_), and D (D_173_, V_242_) of the German Rietberg/2014-like viruses. These eight substitutions are unique in comparisons with (i) the closely related H3_seas_N2_seas_ strains (i.e., A/Denmark/129/2005, A/Bayern/4/2006, A/Berlin/2/2006, A/Lyon/636/2006) and demonstrate significant antigenic drift, (ii) all other H3_seas_N2_seas_ in the past 50 years, and (iii) all known Gent/1984-like swH3N2 (Supplementary Figs. 2, 5). The Danish strains which cluster with the Rietberg/2014-like viruses show some sequence heterogeneity; in particular, they differ from the German isolates in two positions (site A: K_144_ vs. N_144_; site B: G_158_ vs. R/N_158_).

## Discussion

### Reassortment events and spill-over infections

**Figure 4:**
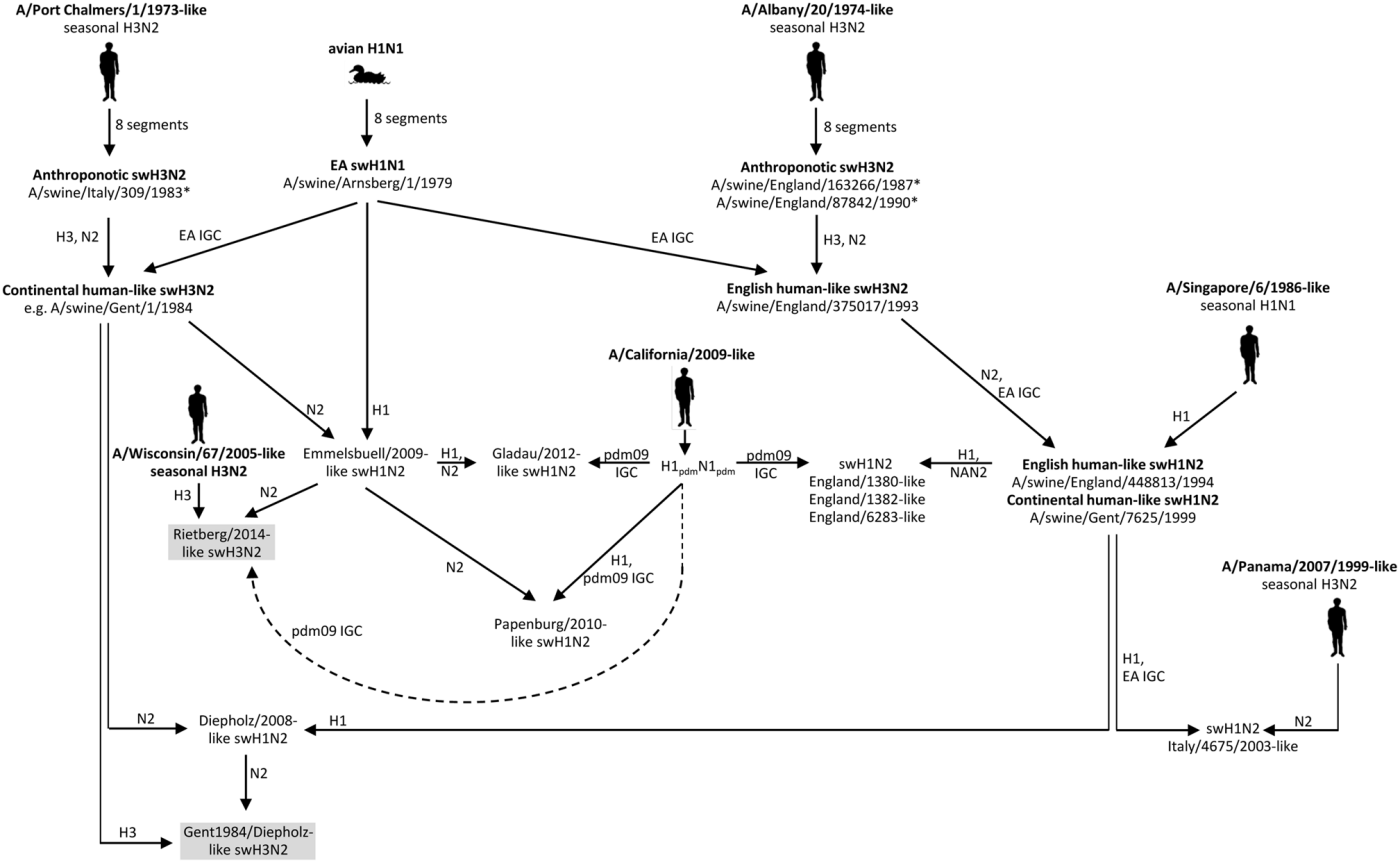
Evolution of German IAV-S. Reassortment events leading to the main IAV-S lineages and the novel swH3N2 viruses (presented in gray boxes) are shown. Pictograms denote anthroponotic and spill-over infections. Asterisks (*) indicate isolates with partial sequences.

Evolution of European IAV-Ss including the novel reassortants is depicted in Fig. 4. The phylogenetic analyses of the HA and NA segments indicate that the continental swH3N2 viruses (genotype #1) and the English swH3N2 viruses (#11) emerged independently from each other, first the continental swH3N2 (Gent/1984-like swH3N2) in 1984, then the English swH3N2 in 1993. Whereas the latter viruses disappeared after a few years, the Gent/1984-like viruses became prevalent in several countries. After many years of circulation, Gent/1984-like viruses faded-out in Germany shortly after appearance of the Gent1984/Diepholz reassortant swH3N2 (#4) which showed a considerably reduced cross-reactivity to the Gent/1984-like swH3N2 (e.g., A/swine/Bakum/IDT1769/2003, Table 1). These viruses have also been detected in the Netherlands, Belgium and France.

Analysis of 228 swH3N2 and six swH3N1 isolates revealed 14 different genome layouts (Fig. 1b) and more than twenty reassortment events (Supplementary Fig. 1, right panel), two of which became established in the German pig population and founded the Rietberg/2014-like swH3N2 viruses and the Gent1984/Diepholz-like swH3N2 (Fig. 4). Superficially, the previously prevalent Gent/1984-like swH3N2 and the Gent1984/Diepholz-like swH3N2 have an identical HA/NA gene constellation, the latter viruses, however, formally resulted from a reassortment including a Gent/1984-like swH3N2 (donor of HA) and a Diepholz/2008-like swH1N2 (donor of NA) and a third, unknown donor of the IGC. This is concluded from the NA tree topology and the phylogenetic analyses of the internal genes (Fig. 2b, Supplementary Figs. 3, 4). Monophyly of all gene segments of the Gent1984/Diepholz reassortant is strong evidence of a single event and cannot explained with another mechanism like genetic drift. The origin of the IGC could not be identified beyond doubt but is very likely of EA swH1N1 origin. Emergence and upsurge of Gent1984/Diepholz reassortant swH3N2 in Germany is associated with the coincident disappearance of the parental Gent/1984-like swH3N2. This exchange has occurred in a period of only two months. In addition to the emergence of reassortants, antigenic drift was observed (Table 1). In particular three substitutions of antigenic site B may have contributed to reduced hemagglutination inhibition of the 2014/15 strains. It is possible that the evolution in antigenic drift of Gent/1984-like swH3N2 and its successor, the Gent1984/Diepholz reassortant, was initiated by the introduction of a trivalent vaccine which was in use in Germany prior to its authorisation since 2008. This vaccine contains a very strong immunogen of the Gent/1984-like swH3N2 lineage.

Besides the reassortment events that led to six persisting swH1N2 and swH3N2 reassortants (Fig. 4), there are six single H3N1 reassortants (#8, #9), three single H3_seas_N2_seas_ reassortants with EA IGC (#13)^9^, six single swH3N2 reassortants with 3A.hu1 HA gene and 2A.hu2b NA gene (#7), and five swH3N2 reassortants with one or more segments of H1_pdm_N1_pdm_ (#2, #3, #5). Altogether, more than 20 events led to swH3N2 or swH3N1 reassortants and have been detected in Germany in the past 30 years (1 event per 18 months). This reassortment frequency is lower than observed for swH1N2^5^ but substantially greater than reassortments of human seasonal IAV. Different reassortment frequencies of human IAVs and IAV-Ss may be explained by two salient factors: (i) Co-circulation of several distinctive IAV-S types and lineages in pigs favours reassortment. In contrast, only two IAV types—H1_pdm_N1_pdm_ and H3_seas_N2_seas_—circulate in the human population and reassortments are rare (e.g., https://www.who.int/csr/don/23-march-2018-seasonal-reassortant-ah1n2-netherlands/en/). (ii) The vast majority of pigs have a short lifespan compared to human longevity. Whereas humans undergo multiple IAV infections that induce a basal immunity to many circulating IAV strains, the majority of the German pig population can be considered as immunologically naïve to most IAV strains due to the short lifespan of fattening pigs. Absence of a broad influenza immunity eases reassortments in herds with concurrent activity of IAV-S and anthroponotic seasonal IAV. The activity of seasonal IAV in pigs is not negligible. Gerhard Elkele's experiments of the early 1930s and their corroboration by Robert Shope and Thomas Francis are early proves of the susceptibility of pigs to human IAVs and until today there are many well-documented examples demonstrating infection of pigs with seasonal IAV followed by virus persistence in the pig population—at least for some time^3,10–12^. The available data indicate that seasonal IAV genes may persist for up to ten years in the gene pool of IAV-Ss without attracting attention. The emergence of the Rietberg/2014-like viruses with seasonal HA gene and pdm09 IGC is another, very recent example. In addition, detection of six anthroponotic swH3N2 isolates (compare Supplementary Fig. 1, left panel) with EA IGC (#10, #14) indicate active exchange between the pools of human and swine influenza viruses. In contrast, there are only few examples of persisting IAV-S circulation in humans. Serological surveys indicated frequent human infections with IAV-S which usually had proceeded unnoticed and without evidence of human-to-human transmission^13,14^. Even so, the Rietberg/2014-like viruses may pose a zoonotic risk as all gene segments of these viruses—directly or indirectly—are of human IAV origin.

### Pig trade and IAV-S dynamics

Germany and Denmark are major pig-producing countries in Europe, each with (i) a highly specialized pig husbandry that has been worked up in the last decades (farrow-to-finish pig farms vs. farrow-to-nursery and finishing farms or farrow-to-wean and wean-to-finish farms), (ii) a high rate of pig trade within the EU that extends country borders, and (iii) a large number of pig holdings. Whereas Denmark is an important exporter of weaners (14.1 million life piglets in 2017), the German pig industry is a major importer of life pigs from Denmark^15^. Recent reports have demonstrated permanently infected farrow-to-finish and farrow-to-nursery pig farms^16,17^ whereas epidemiological models suggest that herd size and a high turnover are drivers of swine influenza persistence^18,19^. Life pig trade links geographically separated pig populations, is believed to support transboundary transmission of IAV-S and may foster virus evolution by reassortment.

The Danish IAV-S epizootiology is characterized by a frequent circulation of EA swH1N1 and reassorted swH1N2 viruses but low prevalence of swH3N2 viruses^1^. This may have supported anthroponotic transmission of seasonal H3N2 viruses from humans to pigs as there was only a low basic immunity against H3 in this pig population. The pdm09 IGC may have provided the ideal components (e.g. characteristically truncated versions of PB1-F2, PA-X and NS1) that allowed to entrench the HA and NA of seasonal H3N2 viruses in a new IAV-S lineage. The first virus of the Rietberg/2014-like viruses was reported from Denmark in 2013^20^. However, sequence data of the internal gene segments have not been published; hence, it is unknown whether this first isolate was already a triple reassortant. In our study, the first virus of this lineage was detected in February 2014 in north-west Germany. Co-circulation in Denmark and Germany and the long persistence suggest a dissemination of newly emerged viruses by life pig trade.

Herd management practices regarding the animal flow and purchase of piglets from various suppliers may influence virus transmission dynamics in all age groups. Beside pig trade, vaccination of sows is another important factor of herd management. Epidemiological models indicate that vaccination may not eliminate swine influenza depending on farm type, herd size and the quality of vaccine-antigen match^21^. The emergence of novel, cocirculating IAV-S variants coincides with increasing herd size but also with the increasing vaccination of pigs in Germany which is striking. It remains to be elucidated whether there is a correlation and how to treat IAV-S dynamics best.

In conclusion, our investigation indicates ongoing IAV-S evolution by reassortment and drift. A more intense IAV-S monitoring and virus sequencing appears to be necessary to improve IAV-S tracing in a timely manner.

## Methods

### Study design

The study was conceptualized as a passive swine influenza survey; details have been published recently^5^. Samples (nasal swabs, serum; bronchoalveolar lavages or lung tissue of perished pigs) were sent in by veterinarians to members of FluResearchNet. Sample collection was performed by veterinarians in accordance with relevant guidelines and regulations and consent for diagnostic PCR, virus isolation and serologic investigation was obtained from participating swine holders. No animal experiments have been performed. Sampling was done as approved by the Landesverwaltungsamt Sachsen-Anhalt for animal experiments of members of the FluResearchNet (Az 42502-3-401, 42502-3-642Ä, 42502-3-743, 45502-3-579).

### Cell lines, virus isolation and virus propagation

Madin–Darby bovine kidney cells (MDBK, ATCC CCL-22) and Madin–Darby canine kidney cells (MDCK, ATCC CCL-34) were maintained in Dulbecco's modified MEM (DMEM) supplemented with 10% fetal bovine serum, 100 U/ml penicillin and 100 µg/ml streptomycin. For virus isolation, PCR-positive samples were used for inoculation of embryonated hens eggs and MDBK cells. All virus isolates were typed by hemagglutination inhibition assays and RT-PCR. For virus propagation, cell culture medium was replaced by serum-free DMEM with 3 µg/ml trypsin and 25 mM MgCl_2_ prior to infection at a multiplicity of infection of 0.01. After complete lysis, supernatants were centrifuged at 1,000 × *g* and aliquoted. Virus stocks were stored at − 80 °C until use.

### Sequence analysis

The genomes of plaque-purified swH3N2 isolates were sequenced either by conventional Sanger sequencing as described previously^22^ or by Illumina sequencing^23^. Total RNA was prepared using the Qiagen RNeasy Mini Kit (Qiagen, Hilden, Germany). Genomic viral RNA (5 µg total RNA) was reverse transcribed using the universal influenza virus primer (5′-RGCRAAAGCAGG-3′) and 20 µl RevertAid premium reverse transcriptase solution (ThermoFisher Scientific, St. Leon-Rot, Germany) following the manufacturer's protocol. Then, cDNA was used for segment-specific amplification; oligonucleotide primers have been described^24^. PCR products were purified and sequenced employing the CEQ DTCS Quick Start Kit (Beckman Coulter, Krefeld, Germany).

Illumina sequencing and de novo assembly was done as described^25^, except sequencing was executed using a GenomeAnalyzer IIx or HiSeq2000/2500. In order to determine the consensus sequence, reads were mapped to reference genomes as described^25^. Assignment to IAV lineages was based on the assembled contigs.

### Phylogenetic analyses

Only complete swH3N2 sequences were used for phylogenetic analyses. In addition to sequence data generated in this study, sequences were retrieved from public databases (GenBank, GISAID) and aligned with MEGA5.2^26^. For alignments, 347 HAH3 sequences and 841 NAN2 sequences were compiled depending on the availability of sequence data. Phylogenetic analyses were conducted with two coalescent tree inference methods (MrBayes, BEAST) using optimal substitution models on the basis of the Bayesian information criterion and the corrected Akaike information criterion^27,28^. The substitution model was selected with the find-best-model option implemented in MEGA.

### Antigenic analysis

Immune sera were established by twofold immunisation of 10 pigs within 21 days (intramuscularly) with 64 hemagglutinating units of inactivated virus A/swine/Bakum/IDT1769/2003 (H3N2) adjuvanted with Carbomer (Carbopol™ 974P NF, Lubrizol). Blood samples were taken 10 days after second administration of the vaccine. Hyper immune sera were established by fourfold immunisation (0, 14, 28, 54 days after first shot) of 1 pig each (intramuscularly) with 64 hemagglutinating units of the corresponding inactivated virus adjuvanted with Freund’s adjuvant (Sigma-Aldrich) or mineral oil (ISA25, Seppic). Blood samples were taken 70 days after first administration of the vaccine. Antibody titers were determined by hemagglutination inhibition (HI) and neutralisation (NT) assays which were performed as described previously^23,29^.

## Supplementary information

Supplementary Information.

## Data Availability

All sequences were submitted to GenBank (acc. nos. MK332395-MK332434, MK361498-MK363253). Supplementary Table 1 summarizes GenBank acc. nos., country and sampling dates of all strains sequenced in the present study.
