# Supplementary material for: Novel reassortant swine H3N2 influenza A viruses in Germany
Source: Sci Rep. 2020 Aug 31;10:14296. doi: 10.1038/s41598-020-71275-5 (PMC7458913; doi:10.1038/s41598-020-71275-5)
Supplement: Supplementary file 1 — Supplementary Information. [file 41598_2020_71275_MOESM1_ESM.pdf]

Supplementary material

Novel reassortant swine H3N2 influenza A viruses in Germany

Roland Zell[1§*] (roland.zell@med.uni-jena.de)
Marco Groth
Andi Krumbholz
Jeannette Lange
Anja Philipps
Ralf Dürrwald

Legends to Supplementary Figures

Supplementary Figure 1: Phylogenetic analysis of HA sequences and genotypes of 341 H3N2/H3N1 strains (lineage H3.seas) and six avian and equine reference strains. The phylogenetic tree was infered with MrBayes; substitution model: GTR+G+I; convergence was reached after 2.4 million generations. The phylogenetic tree (left panel) presents strain designations and GenBank/EpiFlu accession numbers. Colour code of the tree: green, $H3_{seas}N2_{seas}$ (lineage H3.seas); red, Gent/1984-like continental swH3N2 strains (sublineage H3.hu1); purple, English swH3N2 (sublineage H3.hu2); magenta, Rietberg/2014-like swH3N2 (sublineage H3.hu3); light blue, Gent1984/Diepholz-like swH3N2; brown, reference sequences. Squares (■) indicate anthroponotic (reverse zoonotic) strains. The right panel presents the genotypes of the strains and indicates lineages and sublineages. Designation of NA according to Zell et al. (2020); nomenclature of HA sublineages is proposed in this study; EA, internal gene cassette was derived from the European avian (EA) swH1N1.

Supplementary Figure 2: Phylogenetic analysis of 139 HAH3 sequences of lineage 3A (98 $H3_{seas}N2_{seas}$ strains retrieved from the GenBank and EpiFlu databases, 35 swH3N2 isolates) and six reference sequences of other lineages as indicated. GenBank accession numbers and EpiFlu isolate numbers, respectively, and strain designations are presented. Nomenclature of the $H3_{seas}N2_{seas}$ strain subgrouping followed the Worldwide Influenza Centre–WHO Collaborating Centre for Reference and Research on Influenza–The Francis Crick Institute. Colour code: green, $H3_{seas}N2_{seas}$; black, swH3N2 isolates; blue, avian reference strains of various lineages; red, equine reference strain. Analysis of antigenic sites according to Wiley et al. (1981), Smith et al. (2004), Nakajima et al. (2003).

Supplementary Figure 3: Phylogenetic analysis of 841 NAN2 sequences using MrBayes. Substitution model: GTR+G+I; convergence was reached after 6 million generations. The phylogenetic tree presents strain designations and GenBank accession numbers. Colour code of the tree: brown, avian reference sequences; green, $H3_{seas}N2_{seas}$ sequences (HA lineage H3.seas, NA lineage 2A.seas); red, Gent/1984-like continental swH3N2 sequences (HA lineage H3.hu1, NA lineage 2A.hu1a); purple, English human-like swH3N2 sequences (HA sublineage H3.hu2, NA sublineage 2A.hu2); magenta, Rietberg/2014-like swH3N2 sequences (HA sublineage H3.hu3, NA sublineage 2A.hu1b); blue, swH1N2 sequences of various NA sublineages; light blue, Gent1984/Diepholz-like swH3N2 sequences (HA sublineage H3.hu1, NA sublineage 2A.hu1a). Brackets to the right of the tree present designations of virus groups (in colour) and NA (sub-)lineage designations (in black).

Supplementary Figure 4: Phylogenetic tree inference using MrBayes and alignments comprising 1229-1253 sequences of the internal gene segments of IAV. The GTR+G+I substitution model was used. The phylogenetic trees present strain designations and the accession numbers of the GenBank/EpiFlu databases. Colour code of the tree: black, avian, equine and human reference sequences; gray, classical swine (CS) H1N1 and north American triple reassortant (NTR) H1N1 strains; green, Eurasian avian (EA) swH1N1; red, swH3N2; blue, swH1N2; ochre, swH3N1 reassortants; purple, $H1_{pdm}N1_{pdm}$; magenta, reassortants with segments of the $H1_{pdm}N1_{pdm}$. **(A)** 1249 PB2 sequences; convergence was reached after 7 million generations. **(B)** 1253 PB1 sequences; convergence was reached after 11 million generations. *Panel (a)* Tree presenting subtypes, the novel swH1N2 reassortants and few country-specific clades. *Panel (b)* Phylogenetic tree indicating sequences with full-length (90-aa) PB1-F2 (black) and truncated PB1-F2 (red). **(C)** 1269 PA sequences; convergence was reached after 33 million generations. *Panel (a)* Tree presenting subtypes, the novel swH1N2 reassortants and few country-specific clades. *Panel (b)* Phylogenetic tree indicating sequences with full-length PA-X (black) and truncated PA-X (red). **(D)** 1232 NP sequences;

convergence was reached after 30 million generations. **(E)** 1229 M sequences; convergence was reached after 13 million generations. A red arrow indicates the branch with the M2 S31→N substitution leading to amantadine resistance. **(F)** 1249 NS sequences; convergence was reached after 9 million generations. *Panel (a)* Tree presenting subtypes, the novel swH1N2 reassortants and few country-specific clades. *Panel (b)* Phylogenetic tree indicating sequences with full-length NS1 protein (black) and truncated NS1 protein (red).

Supplementary Figure 5: Phylogenetic analysis and antigenic sites of 341 swH3N2/swH3N1 strains (lineage 3A, Lu-2007 nomenclature) and six reference strains. The phylogenetic tree (left panel) is identical to that of Suppl. Fig. 1. Colour code of the tree: green, $H3_{seas}N2_{seas}$ (lineage H3.seas); red, Gent/1984-like continental swH3N2 strains (sublineage H3.hu1); purple, English swH3N2 (sublineage H3.hu2); magenta, Rietberg/2014-like swH3N2 (sublineage H3.hu3); light blue, Gent1984/Diepholz-like swH3N2; brown, reference sequences. Squares (■) indicate anthroponotic (reverse zoonotic) strains. Sublineages and lineages are indicated to the right of the phylogenetic tree. Arrows indicate branches with identical amino acid exchanges. The right panel presents the antigenic sites of the strains. The five antigenic sites were analysed individually. Each amino acid is indicated in a different colour. **Site A:** aa 122, 124, 131, 133, 135, 137, 138, 140-146 of HA; **site B:** aa 155-160, 163, 164, 187-197; **site C:** aa 53, 54, 275-278; **site D:** aa 172-174, 201, 207, 213, 217, 225-230, 242-244; **site E:** aa 62, 63, 78, 82, 83, 260-262 as defined by Wiley et al. (1981), Smith et al. (2004), Nakajima et al. (2003).

Nakajima K, Nobusawa E, Tonegawa E, Nakajima S. 2003. Restriction of amino acid change in influenza A virus H3HA: Comparison of amino acid changes observed in nature and in vitro. J Virol 77(18):10088-10098.

Smith DJ, Lapedes AS, de Jong JC, Bestebroer TM, Rimmelzwaan GF, Osterhaus ADME, Fouchier RAM. 2004. Mapping the antigenic and genetic evolution of influenza virus. Science 305:371-376.

Wiley DC, Wilson IA, Skehel JJ. 1981. Structural identification of the antibody-binding sites of Hong Kong influenza haemagglutinin and their involvement in antigenic variation. Nature 289:373-378.

Zell R, Groth M, Krumbholz A, Lange J, Philipps A, Dürrwald R. 2020 Displacement of the Gent/1999 human-like swine H1N2 influenza A virus lineage by novel H1n2 reassortants in Germany. Arch Virol 165:55-67.

Suppl. Fig. 1

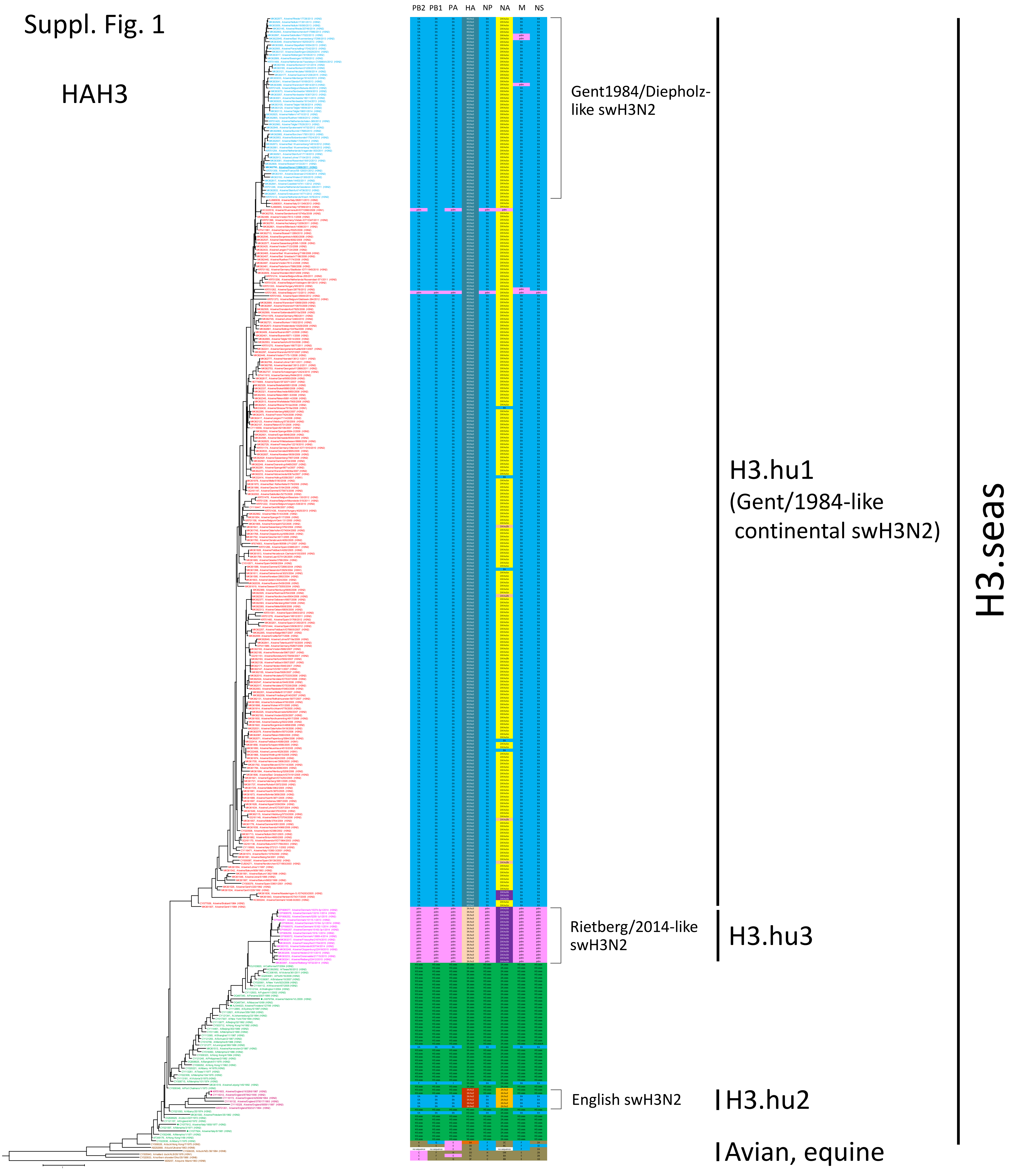
HAH3
PB2
PB1
PA
HA
NP
NA
M
NS
Gent1984/Diepholz-like swH3N2
H3.hu1
(Gent/1984-like continental swH3N2)
Rietberg/2014-like swH3N2
H3.hu3
English swH3N2
H3.hu2
Avian, equine
H3.seas

Suppl. Fig. 2

HAH3

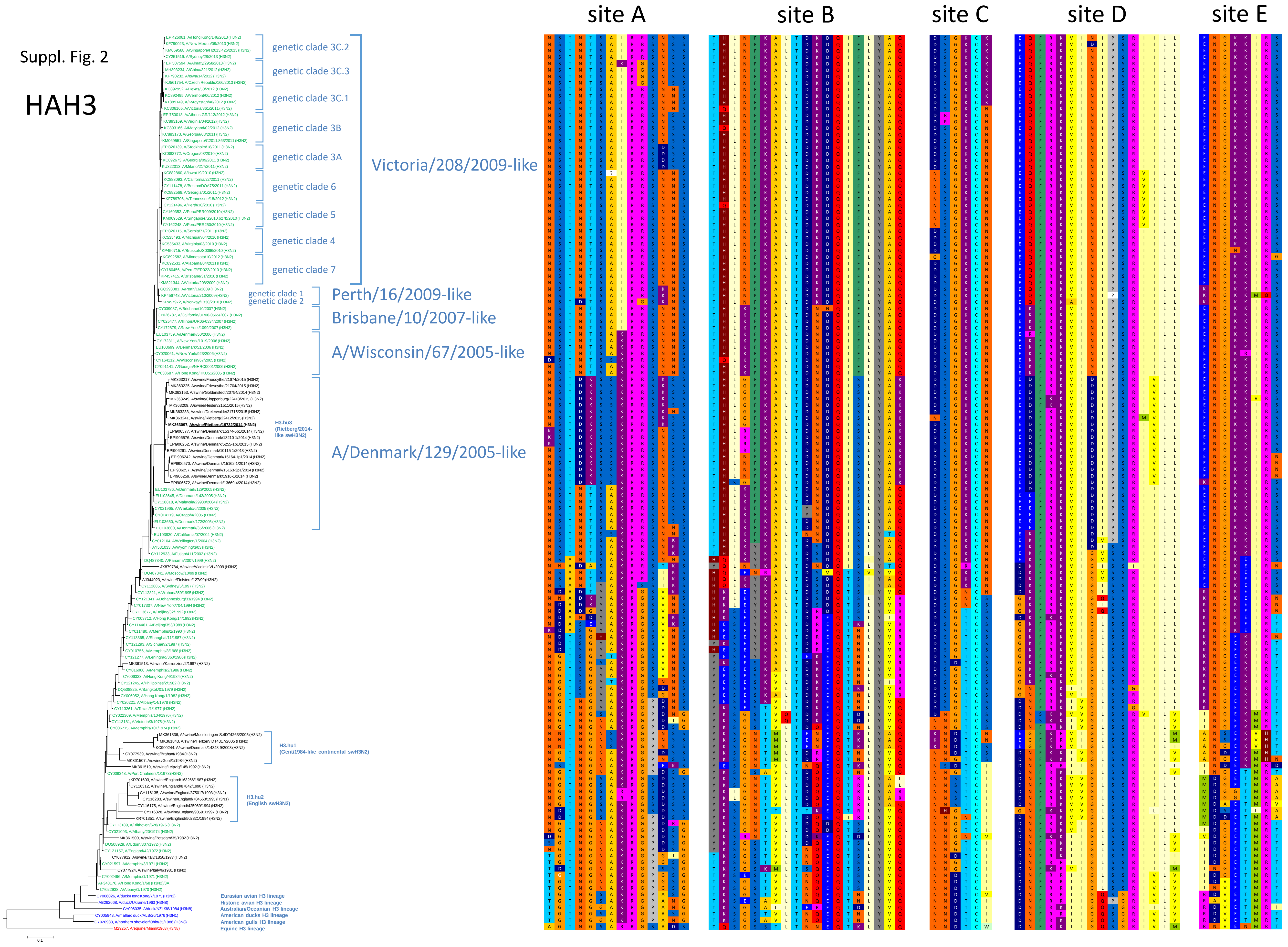

Suppl. Fig. 3

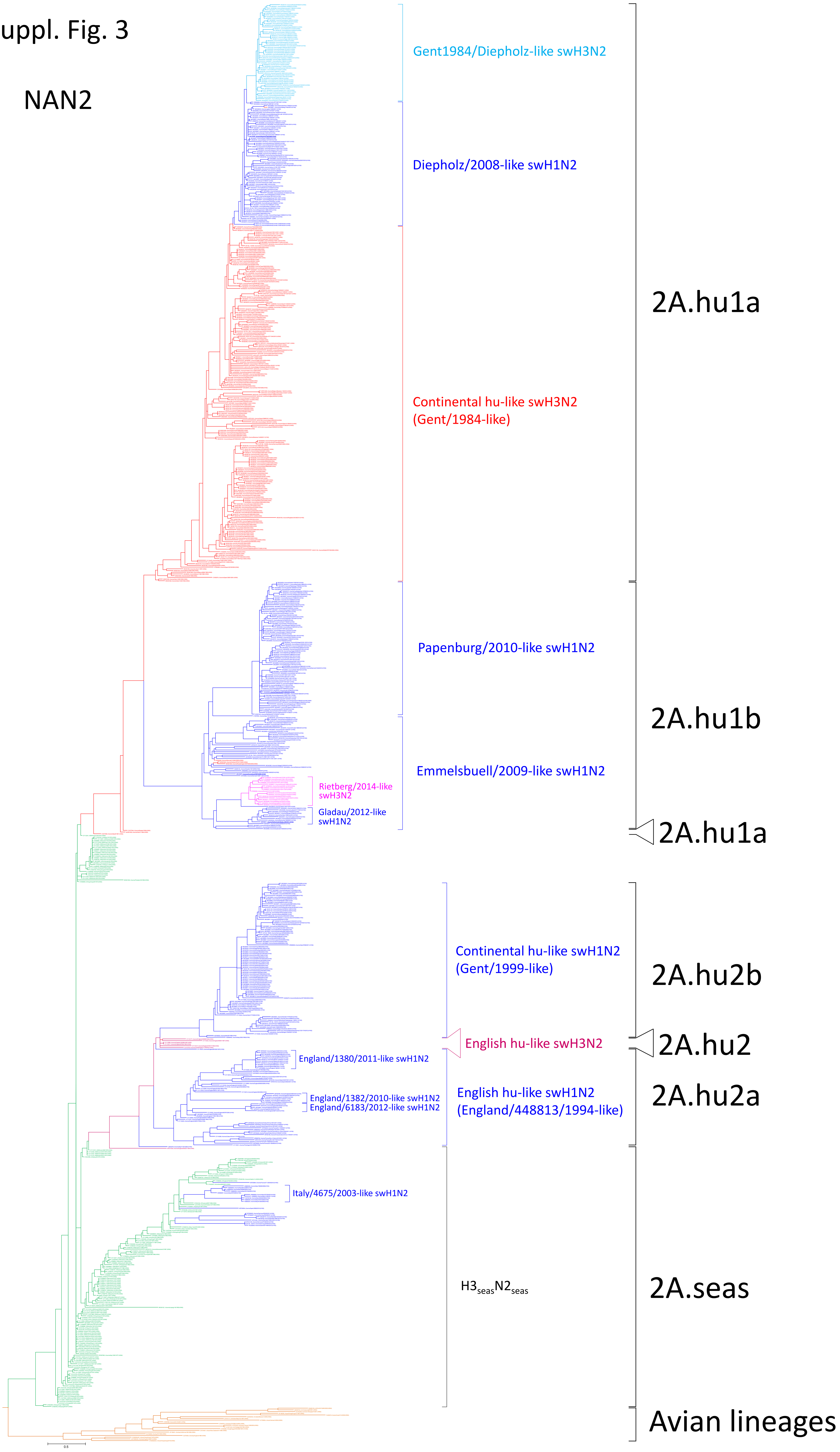
NAN2
Gent1984/Diepholz-like swH3N2
Diepholz/2008-like swH1N2
Continental hu-like swH3N2
(Gent/1984-like)
2A.hu1a
Papenburg/2010-like swH1N2
Emmelsbuell/2009-like swH1N2
Rietberg/2014-like
swH3N2
Gladau/2012-like
swH1N2
2A.hu1b
2A.hu1a
Continental hu-like swH1N2
(Gent/1999-like)
2A.hu2b
English hu-like swH3N2
2A.hu2
England/1380/2011-like swH1N2
England/1382/2010-like swH1N2
England/6183/2012-like swH1N2
English hu-like swH1N2
(England/448813/1994-like)
2A.hu2a
Italy/4675/2003-like swH1N2
$H3_{seas}N2_{seas}$
2A.seas
Avian lineages
0.5

Suppl. Fig. 4A

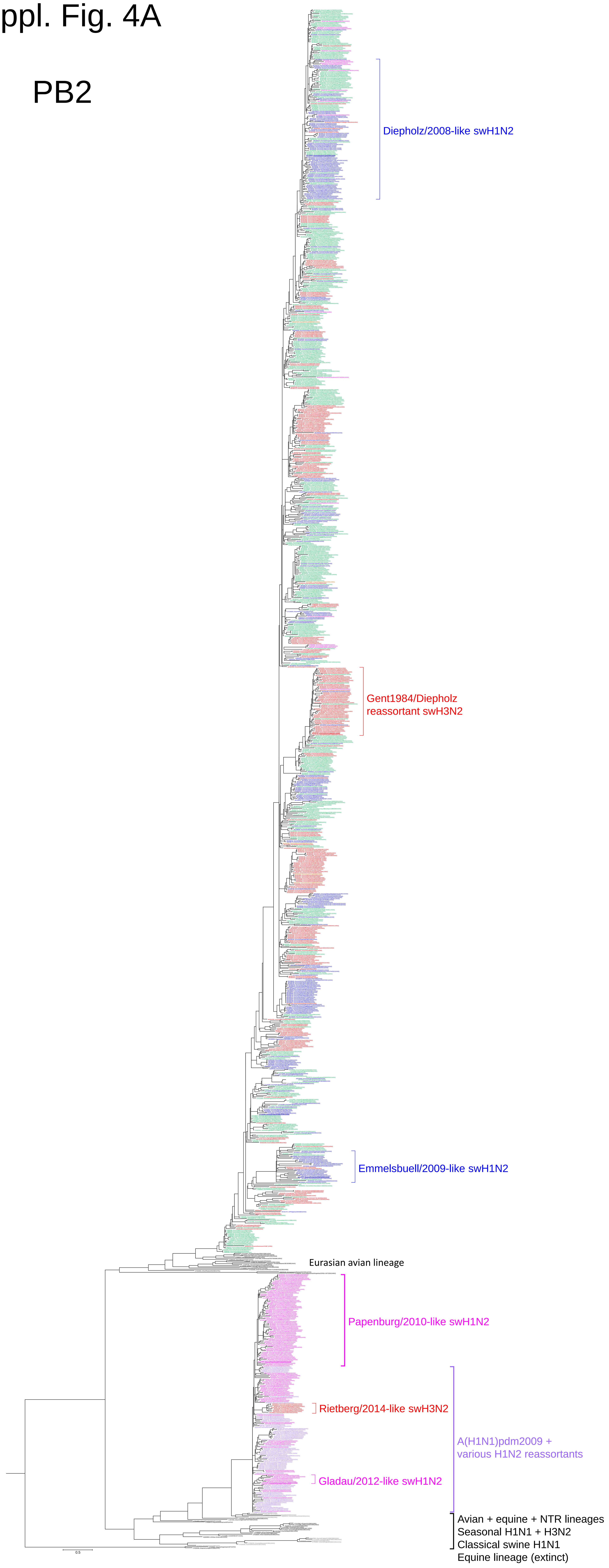
PB2
Diepholz/2008-like swH1N2
Gent1984/Diepholz
reassortant swH3N2
Emmelsbuell/2009-like swH1N2
Eurasian avian lineage
Papenburg/2010-like swH1N2
Rietberg/2014-like swH3N2
A(H1N1)pdm2009 +
various H1N2 reassortants
Gladau/2012-like swH1N2
Avian + equine + NTR lineages
Seasonal H1N1 + H3N2
Classical swine H1N1
Equine lineage (extinct)
0.5

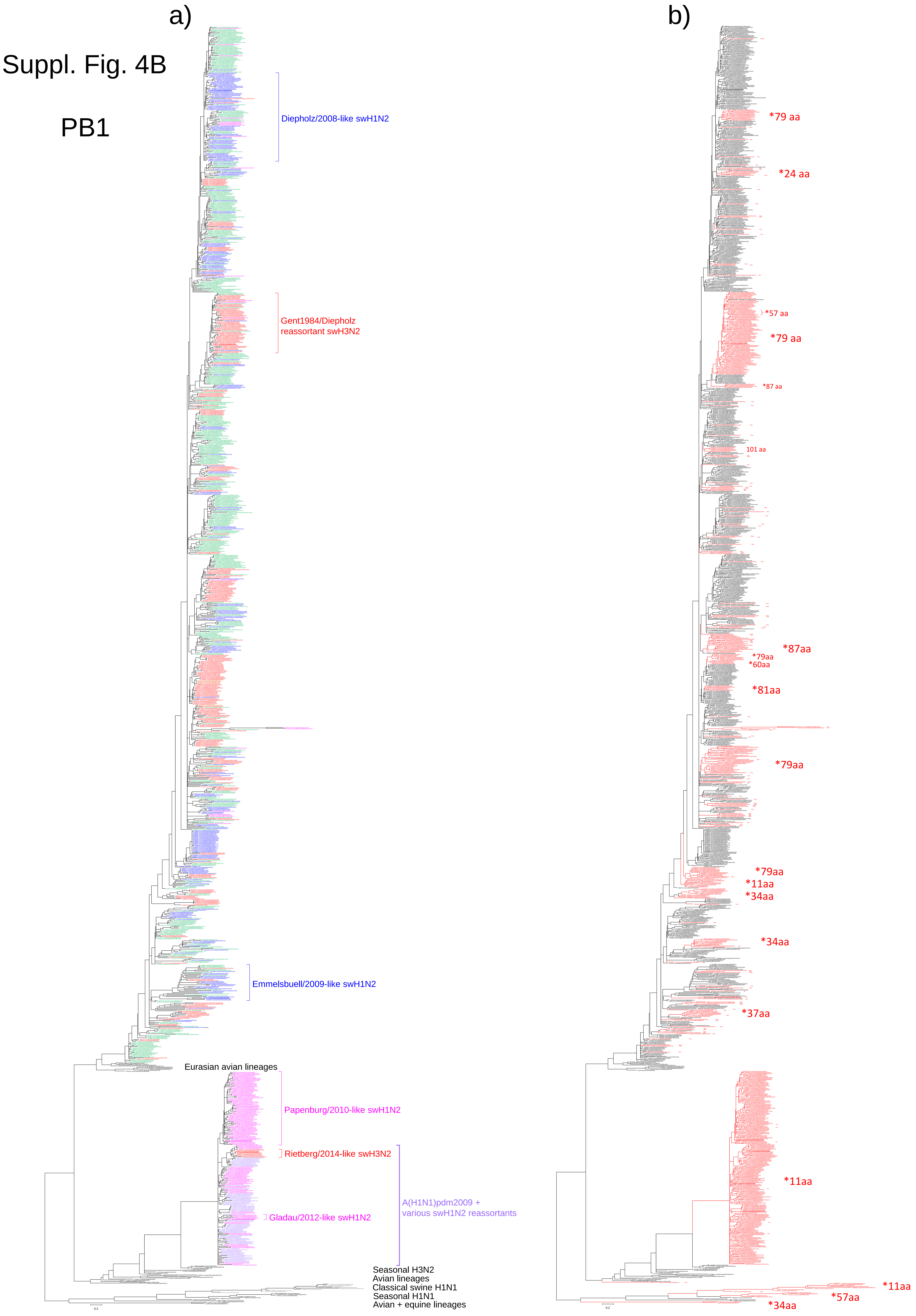
Suppl. Fig. 4B
PB1
a)
b)
Diepholz/2008-like swH1N2
Gent1984/Diepholz
reassortant swH3N2
Emmelsbuell/2009-like swH1N2
Eurasian avian lineages
Papenburg/2010-like swH1N2
Rietberg/2014-like swH3N2
A(H1N1)pdm2009 +
various swH1N2 reassortants
Gladau/2012-like swH1N2
Seasonal H3N2
Avian lineages
Classical swine H1N1
Seasonal H1N1
Avian + equine lineages
0.2
*79 aa
*24 aa
*57 aa
*79 aa
*87 aa
101 aa
*87aa
*79aa
*60aa
*81aa
*79aa
*79aa
*11aa
*34aa
*34aa
*37aa
*11aa
*11aa
*57aa
*34aa
0.2

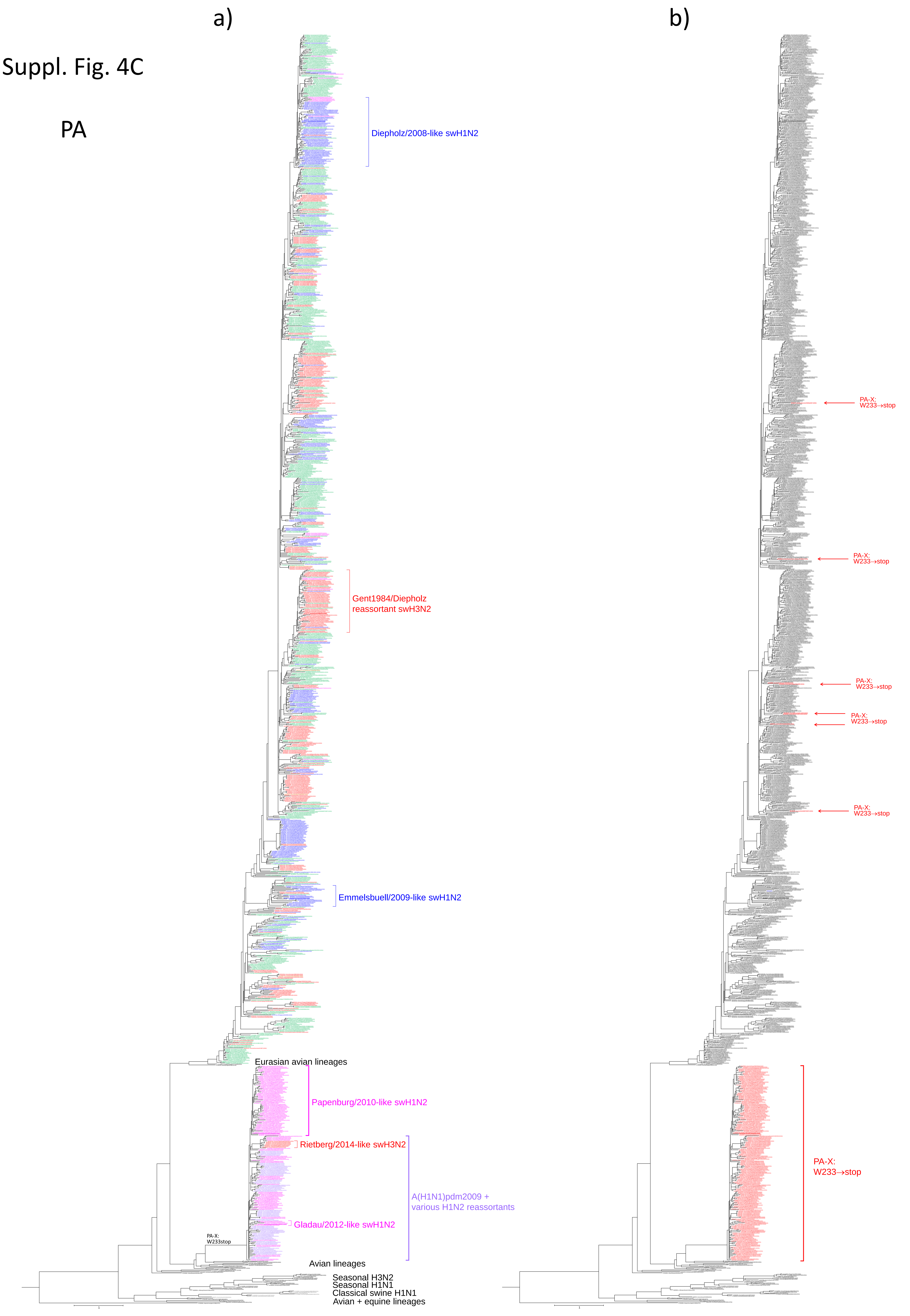

Suppl. Fig. 4C
PA
a)
b)
Diepholz/2008-like swH1N2
Gent1984/Diepholz
reassortant swH3N2
Emmelsbuell/2009-like swH1N2
Eurasian avian lineages
Papenburg/2010-like swH1N2
Rietberg/2014-like swH3N2
A(H1N1)pdm2009 +
various H1N2 reassortants
Gladau/2012-like swH1N2
PA-X:
W233stop
Avian lineages
Seasonal H3N2
Seasonal H1N1
Classical swine H1N1
Avian + equine lineages
PA-X:
W233→stop
PA-X:
W233→stop
PA-X:
W233→stop
PA-X:
W233→stop
PA-X:
W233→stop
PA-X:
W233→stop

Suppl. Fig. 4D

NP

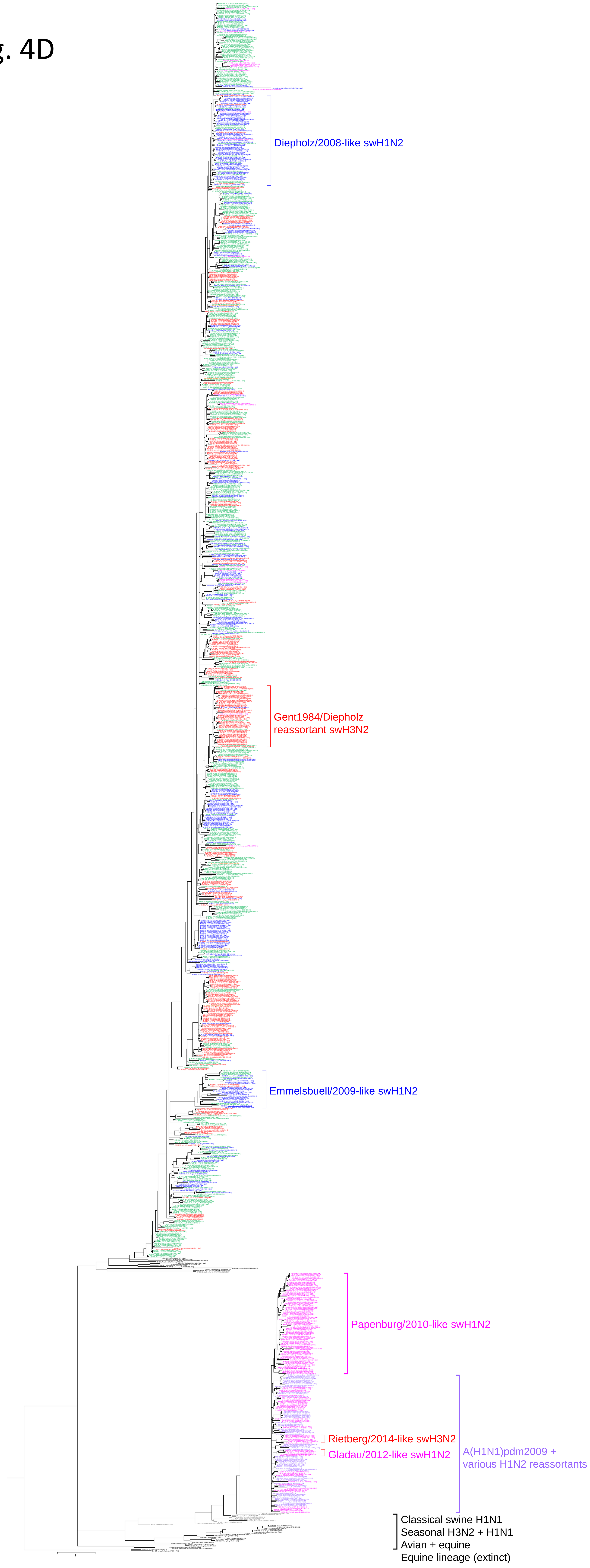

Suppl. Fig. 4E

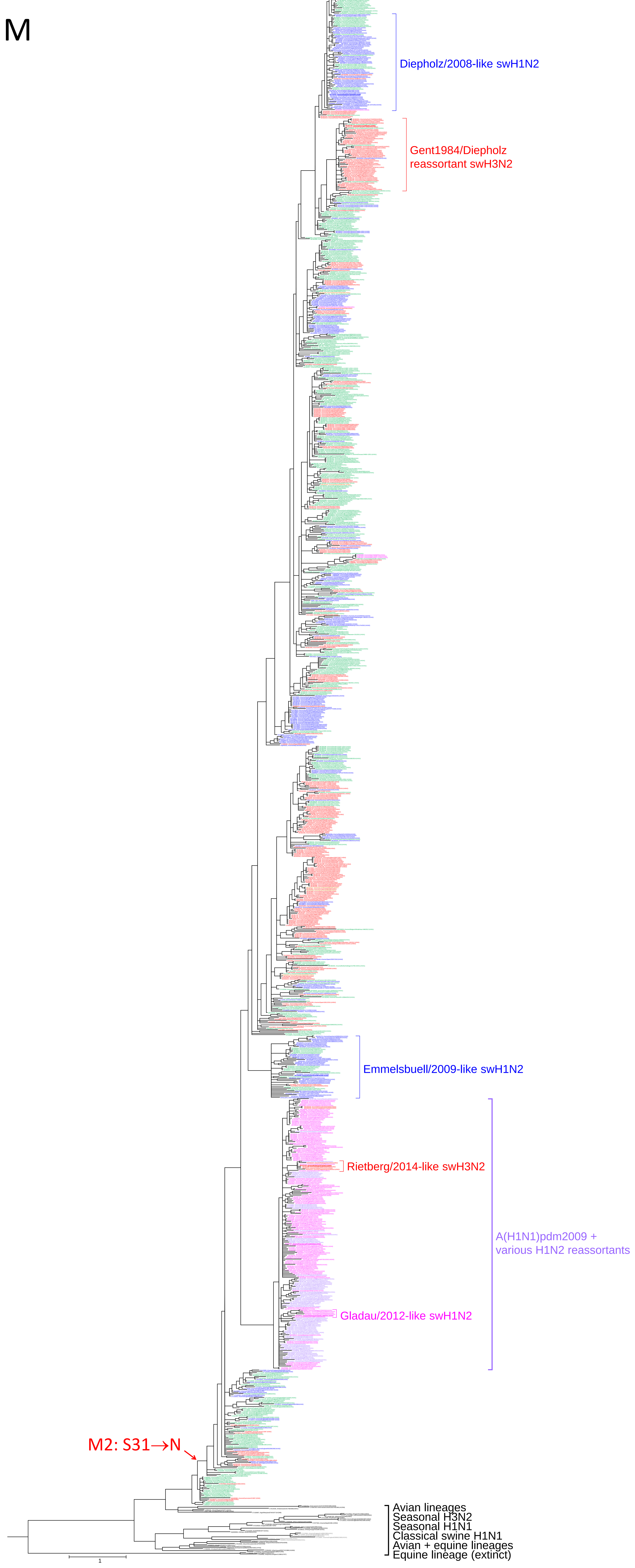
M
Diepholz/2008-like swH1N2
Gent1984/Diepholz
reassortant swH3N2
Emmelsbuell/2009-like swH1N2
Rietberg/2014-like swH3N2
A(H1N1)pdm2009 +
various H1N2 reassortants
Gladau/2012-like swH1N2
M2: S31→N
Avian lineages
Seasonal H3N2
Seasonal H1N1
Classical swine H1N1
Avian + equine lineages
Equine lineage (extinct)
1

Suppl. Fig. 4F,
NS

a)

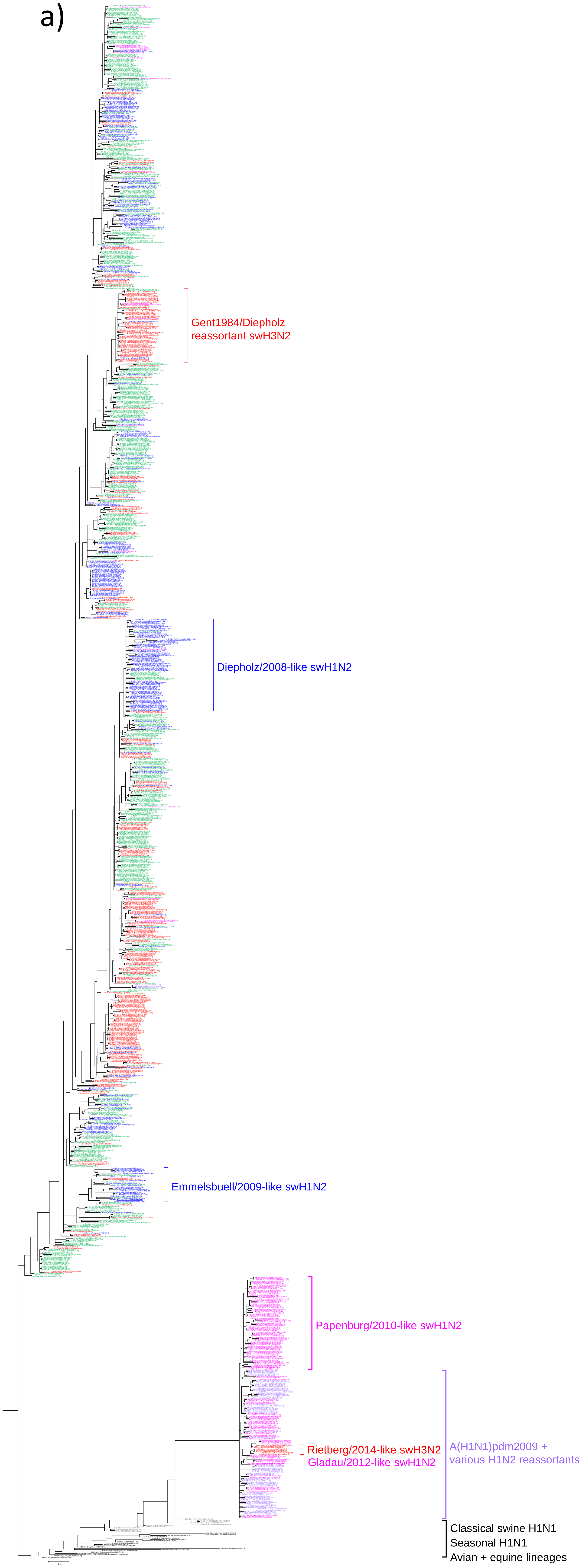

b)

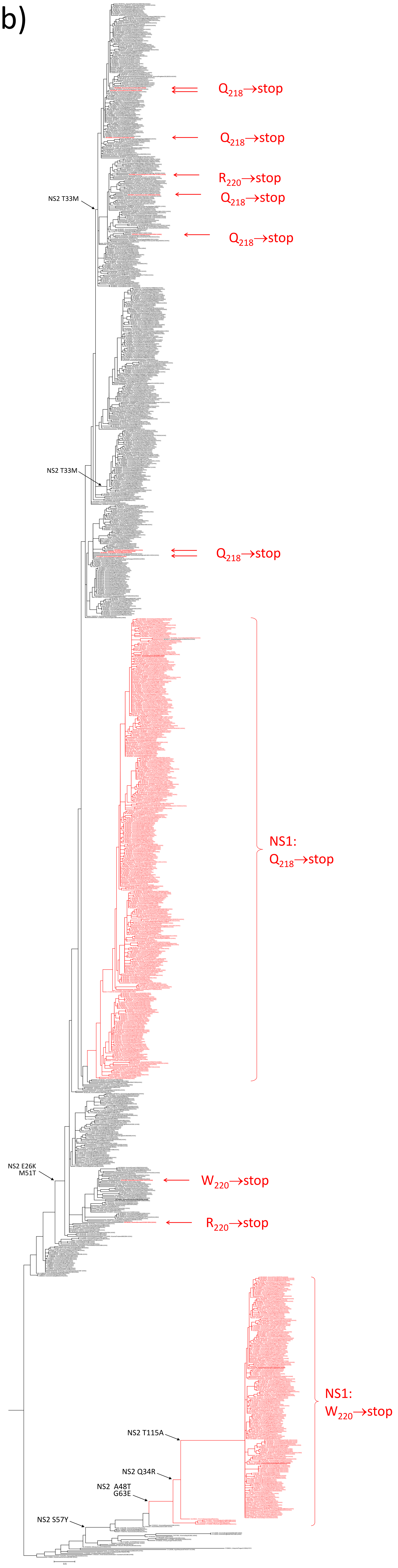

Suppl. Fig. 5

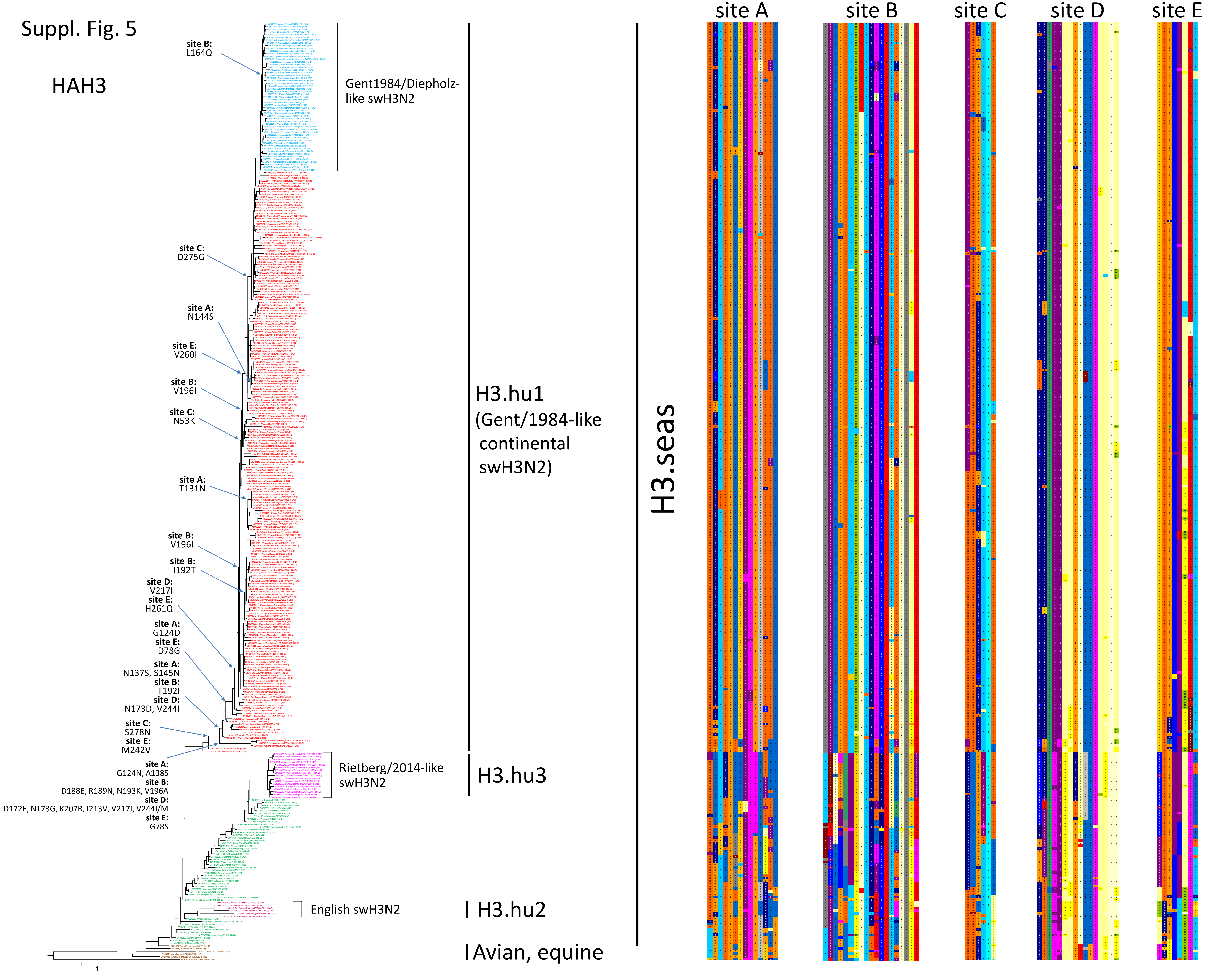
HAH3
site B:
L164Q
Gent1984/Diepholz-like swH3N2
site C:
D275G
site A:
N144S
site E:
V260I
site B:
V196I
site C:
N53K
site A:
T131N
site B:
V196I
site B:
I192T
site D:
V217I
site E:
H261Q
site A:
G124D
site E:
D78G
site A:
N137S, S145N
site B:
T192I
site D:
N173D, V244I
site C:
S278N
site E:
M242V
site A:
G124N, A138S
site B:
D188E, R189N, N193K, V196A
site D:
D172E, N173G, K207R, I213V, V217I, V244I/M
site E:
G78S
Rietberg/2014-like swH3N2
English swH3N2
H3.hu1
(Gent/1984-like continental swH3N2)
H3.hu3
H3.hu2
Avian, equine
H3.seas
site A
site B
site C
site D
site E
1

Strain	Sampling date	Country	Seg. 1, Pol PB2	Seg. 2, Pol PB1	Seg. 3, Pol PA	Seg. 4, HA	Seg. 5, NP	Seg. 6, NA	Seg. 7, M	Seg. 8, NS
A/swine/Potsdam/35/1982 (H3N2)	1982	Germany	MK361498	DQ836171	MK361499	MK361500	MK361501	MK361502	DQ186979	MK361503
A/swine/Gent/1/1984 (H3N2)	1984	Belgium	MK361504	MK361505	MK361506	MK361507	MK361508	MK361509	EU478798	MK361510
A/swine/Karrenzien/2/1987 (H3N2)	1987	Germany	MK361511	DQ836169	MK361512	MK361513	MK361514	MK361515	DQ186980	MK361516
A/swine/Leipzig/145/1992 (H3N2)	1992	Germany	MK361517	DQ836173	MK361518	MK361519	MK361520	MK361521	DQ186981	MK361522
A/swine/Gent/V220/1992 (H3N2)	1992	Belgium	MK361523	MK361524	MK361525	MK361526	MK361527	MK361528	MK361529	MK361530
A/swine/Gent/V229/1992 (H3N2)	1992	Belgium	MK361531	MK361532	MK361533	MK361534	MK361535	MK361536	MK361537	MK361538
A/swine/Bakum/909/1993 (H3N2)	1993	Germany	MK361539	MK361540	MK361541	MK361542	MK361543	MK361544	EU478801	MK361545
A/swine/Jena/5/1996 (H3N2)	1996	Germany	MK361546	DQ836168	MK361547	MK361548	MK361549	MK361550	DQ186982	MK361551
A/swine/Lohne/1/1997 (H3N2)	1997	Germany	MK361552	DQ836172	MK361553	MK361554	MK361555	MK361556	DQ186983	MK361557
A/swine/Bakum/1362/1998 (H3N2)	1998	Germany	MK361558	MK361559	MK361560	MK361561	MK361562	MK362563	EU478803	MK361564
A/swine/Bakum/8602/1999 (H3N2)	1999	Germany	MK361565	DQ836170	MK361566	MK361567	MK361568	MK361569	DQ186984	MK361570
A/swine/Berlin/1578/2000 (H3N2)	2000	Germany	MK361571	MK361572	MK361573	MK361574	MK361575	MK361576	EU478805	MK361577
A/swine/Belzig/54/2001 (H3N2)	2001	Germany	MK361578	MK361579	MK361580	MK361581	MK361582	MK361583	EU478806	MK361584
A/swine/Bakum/IDT1769-Pass1/2003 (H3N2)	07-Mar-2003	Germany	GQ161133	GQ161134	GQ161135	GQ161136	GQ161137	EU163948	EU478807	GQ161138
A/swine/Bakum/IDT1769-Pass22/2003 (H3N2)	07-Mar-2003	Germany	GQ161126	GQ161127	GQ161128	GQ161129	GQ161130	GQ161100	GQ161131	GQ161132
A/swine/Bissendorf/IDT1864/2003 (H3N2)	19-Mar-2004	Germany	GQ161167	GQ161168	GQ161169	GQ161170	GQ161171	GQ161172	EU478808	GQ161173
A/swine/Nordkirchen/IDT1993/2003 (H3N2)	09-Apr-2003	Germany	EU924268	EU924269	EU924270	EU924271	EU924272	EU924273	EU478814	EU924274
A/swine/Damme/IDT2890/2004 (H3N2)	02-Jan-2004	Germany	MK361585	MK361586	MK361587	MK361588	MK361589	MK361590	EU478820	MK361591
A/swine/Kevelaer/2892/2004 (H3N2)	06-Jan-2004	Germany	MK361592	MK361593	MK361594	MK361595	MK361596	MK361597	MK361598	MK361599
A/swine/Uedem/3024/2004 (H3N2)	12-Mar-2004	Germany	MK361600	MK361601	MK361602	MK361603	MK361604	MK361605	MK361606	MK361607
A/swine/Delmenhorst/3025/2004 (H3N2)	11-Mar-2004	Germany	MK361608	MK361609	MK361610	MK361611	MK361612	MK361613	MK361614	MK361615
A/swine/Seesen/IDT3055/2004 (H3N2)	15-Mar-2004	Germany	MK361616	MK361617	MK361618	MK361619	MK361620	MK361621	EU478828	MK361622
A/swine/Appel/3339/2004 (H3N2)	05-Jul-2004	Germany	MK361623	MK361624	MK361625	MK361626	MK361627	MK361628	MK361629	MK361630
A/swine/Lohne/IDT3357/2004 (H3N2)	26-Jul-2004	Germany	MK361631	MK361632	MK361633	MK361634	MK361635	MK361636	EU478826	MK361637
A/swine/Sassenberg/3762/2004 (H3N2)	17-Dec-2004	Germany	MK361638	MK361639	MK361640	MK361641	MK361642	MK361643	MK361644	MK361645
A/swine/Hörstel/3763/2004 (H3N2)	06-Dec-2004	Germany	MK361646	MK361647	MK361648	MK361649	MK361650	MK361651	MK361652	MK361653
A/swine/Melle/3764/2004 (H3N2)	18-Dec-2004	Germany	MK361654	MK361655	MK361656	MK361657	MK361658	MK361659	MK361660	MK361661
A/swine/Geseke/3766/2004 (H3N2)	22-Dec-2004	Germany	MK361662	MK361663	MK361664	MK361665	MK361666	MK361667	MK361668	MK361669
A/swine/Bohmte/3856/2005 (H3N2)	03-Feb-2005	Germany	MK361670	MK361671	MK361672	MK361673	MK361674	MK361675	MK361676	MK361677
A/swine/Hürth/3870/2005 (H3N2)	02-Feb-2005	Germany	MK361678	MK361679	MK361680	MK361681	MK361682	MK361683	MK361684	MK361685
A/swine/Hürth/3871/2005 (H3N2)	02-Feb-2005	Germany	MK361686	MK361687	MK361688	MK361689	MK361690	MK361691	MK361692	MK361693
A/swine/Grebenau/3887/2005 (H3N2)	15-Feb-2005	Germany	MK361694	MK361695	MK361696	MK361697	MK361698	MK361699	MK361700	MK361701
A/swine/Hannover/3906/2005 (H3N2)	21-Feb-2005	Germany	MK361702	MK361703	MK361704	MK361705	MK361706	MK361707	MK361708	MK361709
A/swine/Nottuln/3921/2005 (H3N2)	25-Feb-2005	Germany	MK361710	MK361711	MK361712	MK361713	MK361714	MK361715	MK361716	MK361717
A/swine/Isterberg/3951/2005 (H3N2)	08-Mar-2005	Germany	MK361718	MK361719	MK361720	MK361721	MK361722	MK361723	MK361724	MK361725
A/swine/Melle/3962/2005 (H3N2)	14-Mar-2005	Germany	MK361726	MK361727	MK361728	MK361729	MK361730	MK361731	MK361732	MK361733
A/swine/Ruhstorf/3972/2005 (H3N2)	17-Mar-2005	Germany	MK361734	MK361735	MK361736	MK361737	MK361738	MK361739	MK361740	MK361741
A/swine/Osterhofen/IDT4004/2005 (H3N2)	31-Mar-2005	Germany	MK361742	MK361743	MK361744	MK361745	MK361746	MK361747	EU478841	MK361748
A/swine/Gescher/4011/2005 (H3N2)	06-Apr-2005	Germany	MK361749	MK361750	MK361751	MK361752	MK361753	MK361754	MK361755	MK361756
A/swine/Osnabrück/4050/2005 (H3N2)	19-Apr-2005	Germany	MK361757	MK361758	MK361759	MK361760	MK361761	MK361762	MK361763	MK361764
A/swine/Cloppenburg/4056/2005 (H3N2)	21-Apr-2005	Germany	MK361765	MK361766	MK361767	MK361768	MK361769	MK361770	MK361771	MK361772
A/swine/Damme/4091/2005 (H3N2)	09-May-2005	Germany	MK361773	MK361774	MK361775	MK361776	MK361777	MK361778	MK361779	MK361780
A/swine/Rhede/4098/2005 (H3N2)	09-May-2005	Germany	MK361781	MK361782	MK361783	MK361784	MK361785	MK361786	MK361787	MK361788
A/swine/Merzen/IDT4114/2005 (H3N2)	07-May-2005	Germany	MK361789	MK361790	MK361791	MK361792	MK361793	MK361794	EU478839	MK361795
A/swine/Laar/IDT4126/2005 (H3N2)	18-May-2005	Germany	MK361796	MK361797	MK361798	MK361799	MK361800	MK361801	EU478838	MK361802
A/swine/Bad Griesbach/IDT4191/2005 (H3N2)	14-Jun-2005	Germany	MK361803	MK361804	MK361805	MK361806	MK361807	MK361808	EU478831	MK361809
A/swine/Herzebrock-Clarholz/4193/2005 (H3N2)	16-Jun-2005	Germany	MK361810	MK361811	MK361812	MK361813	MK361814	MK361815	MK361816	MK361817
A/swine/Egglham/IDT4250/2005 (H3N2)	01-Jul-2005	Germany	MK361818	MK361819	MK361820	MK361821	MK361822	MK361823	EU478833	MK361824
A/swine/Feldbach/4262/2005 (H3N2)	08-Jul-2005	Austria	MK361825	MK361826	MK361827	MK361828	MK361829	MK361830	MK361831	MK361832
A/swine/Muesleringen-S./IDT4263/2005 (H3N2)	11-Jul-2005	Germany	MK361833	MK361834	MK361835	MK361836	MK361837	MK361838	EU478840	MK361839
A/swine/Hertzen/IDT4317/2005 (H3N2)	22-Jul-2005	Germany	MK361840	MK361841	MK361842	MK361843	MK361844	MK361845	EU478835	MK361846
A/swine/Neuenhaus/4515/2005 (H3N2)	30-Sep-2005	Germany	MK361847	MK361848	MK361849	MK361850	MK361851	MK361852	MK361853	MK361854
A/swine/Schapen/4566/2005 (H3N2)	17-Oct-2005	Germany	MK361855	MK361856	MK361857	MK361858	MK361859	MK361860	MK361861	MK361862
A/swine/Wettrup/4615/2005 (H3N2)	31-Oct-2005	Germany	MK361863	MK361864	MK361865	MK361866	MK361867	MK361868	MK361869	MK361870
A/swine/Elze/4624/2005 (H3N2)	02-Nov-2005	Germany	MK361871	MK361872	MK361873	MK361874	MK361875	MK361876	MK361877	MK361878
A/swine/Brilon/4685/2005 (H3N2)	25-Nov-2005	Germany	MK361879	MK361880	MK361881	MK361882	MK361883	MK361884	MK361885	MK361886
A/swine/Schnaitsee/4700/2005 (H3N2)	28-Nov-2005	Germany	MK361887	MK361888	MK361889	MK361890	MK361891	MK361892	MK361893	MK361894
A/swine/Welver/4701/2005 (H3N2)	30-Nov-2005	Germany	MK361895	MK361896	MK361897	MK361898	MK361899	MK361900	MK361901	MK361902
A/swine/Krempel/4722/2005 (H3N2)	06-Dec-2005	Germany	MK361903	MK361904	MK361905	MK361906	MK361907	MK361908	MK361909	MK361910
A/swine/Kirchham/4778/2005 (H3N2)	16-Dec-2005	Germany	MK361911	MK361912	MK361913	MK361914	MK361915	MK361916	MK361917	MK361918
A/swine/Borgentreich/4858/2006 (H3N2)	13-Jan-2006	Germany	MK361919	MK361920	MK361921	MK361922	MK361923	MK361924	MK361925	MK361926
A/swine/Nordhümmling/4917/2006 (H3N2)	27-Jan-2006	Germany	MK361927	MK361928	MK361929	MK361930	MK361931	MK361932	MK361933	MK361934
A/swine/Asendorf/4966/2006 (H3N2)	02-Feb-2006	Germany	MK361935	MK361936	MK361937	MK361938	MK361939	MK361940	MK361941	MK361942
A/swine/Daseburg/5022/2006 (H3N2)	24-Feb-2006	Germany	MK361943	MK361944	MK361945	MK361946	MK361947	MK361948	MK361949	MK361950
A/swine/Spenge/5117/2006 (H3N2)	30-Mar-2006	Germany	MK361951	MK361952	MK361953	MK361954	MK361955	MK361956	MK361957	MK361958
A/swine/Hilter/5143/2006 (H3N2)	10-Apr-2006	Germany	MK361959	MK361960	MK361961	MK361962	MK361963	MK361964	MK361965	MK361966
A/swine/Bad Rothenfelde/5179/2006 (H3N2)	24-Apr-2006	Germany	MK361967	MK361968	MK361969	MK361970	MK361971	MK361972	MK361973	MK361974
A/swine/Melle/5190/2006 (H3N2)	25-Apr-2006	Germany	MK361975	MK361976	MK361977	MK361978	MK361979	MK361980	MK361981	MK361982
A/swine/Gescher/5194/2006 (H3N2)	27-Apr-2006	Germany	MK361983	MK361984	MK361985	MK361986	MK361987	MK361988	MK361989	MK361990
A/swine/Nienburg/5208/2006 (H3N2)	30-Apr-2006	Germany	MK361991	MK361992	MK361993	MK361994	MK361995	MK361996	MK361997	MK361998
A/swine/Salzkotten/5275/2006 (H3N2)	23-May-2006	Germany	MK361999	MK362000	MK362001	MK362002	MK362003	MK362004	MK362005	MK362006
A/swine/Herzlake/IDT5335/2006 (H3N2)	20-Jun-2006	Germany	MK362007	MK362008	MK362009	MK362010	MK362011	MK362012	EU478847	MK362013
A/swine/Herzlake/IDT5336/2006 (H3N2)	20-Jun-2006	Germany	MK362014	MK362015	MK362016	MK362017	MK362018	MK362019	EU478848	MK362020
A/swine/Herzlake/IDT5337/2006 (H3N2)	20-Jun-2006	Germany	MK362021	MK362022	MK362023	MK362024	MK362025	MK362026	EU478849	MK363027
A/swine/Osterhofen/5418/2006 (H3N2)	25-Jul-2006	Germany	MK362028	MK362029	MK362030	MK362031	MK362032	MK362033	MK362034	MK362035
A/swine/Büren/5439/2006 (H3N2)	02-Aug-2006	Germany	MK362036	MK362037	MK362038	MK362039	MK362040	MK362041	MK362042	MK362043
A/swine/Hamstrub/5445/2006 (H3N2)	03-Aug-2006	Germany	MK362044	MK362045	MK362046	MK362047	MK362048	MK362049	MK362050	MK362051
A/swine/Erwitte/5477/2006 (H3N2)	15-Aug-2006	Germany	MK362052	MK362053	MK362054	MK362055	MK362056	MK362057	MK362058	MK362059

A/swine/Raddestorf/5483/2006 (H3N2)	15-Aug-2006	Germany	MK362060	MK362061	MK362062	MK362063	MK362064	MK362065	MK362066	MK362067
A/swine/Papenburg/5564/2006 (H3N2)	27-Sep-2006	Germany	MK362068	MK362069	MK362070	MK362071	MK362072	MK362073	MK362074	MK362075
A/swine/Stadtlohn/5570/2006 (H3N2)	27-Sep-2006	Germany	MK362076	MK362077	MK362078	MK362079	MK362080	MK362081	MK362082	MK362083
A/swine/Reken/5580/2006 (H3N2)	05-Oct-2006	Germany	MK362084	MK362085	MK362086	MK362087	MK362088	MK362089	MK362090	MK362091
A/swine/Damme/IDT5673/2006 (H3N2)	15-Nov-2006	Germany	MK362092	MK362093	MK362094	GQ161147	MK362095	GQ161148	MK362096	MK362097
A/swine/Melle/IDT5706/2006 (H3N2)	20-Nov-2006	Germany	MK362098	MK362099	MK362100	GQ161149	MK362101	GQ161150	MK362102	MK362103
A/swine/Reken/5731/2006 (H3N2)	27-Nov-2006	Germany	MK362104	MK362105	MK362106	MK362107	MK362108	MK362109	MK362110	MK362111
A/swine/Vilsbiburg/5733/2006 (H3N2)	23-Nov-2006	Germany	MK362112	MK362113	MK362114	MK362115	MK362116	MK362117	MK362118	MK362119
A/swine/Vilsbiburg/5735/2006 (H3N2)	27-Nov-2006	Germany	MK+D57362 120	MK362121	MK362122	MK362123	MK362124	MK362125	MK362126	MK362127
A/swine/Rotthalmünster/5877/2007 (H3N2)	20-Jan-2007	Germany	MK362128	MK362129	MK362130	MK362131	MK362132	MK362133	MK362134	MK362135
A/swine/Feldbach/5907/2007 (H3N2)	06-Feb-2007	Austria	MK362136	MK362137	MK362138	MK362139	MK362140	MK362141	MK362142	MK362143
A/swine/IVD/5911/2007 (H3N2)	05-Feb-2007	Germany	MK362144	MK362145	MK362146	MK362147	MK362148	MK362149	MK362150	MK362151
A/swine/Gnas/5926/2007 (H3N2)	16-Feb-2007	Austria	MK362152	MK362153	MK362154	MK362155	MK362156	MK362157	MK362158	MK362159
A/swine/Herford/5932/2007 (H3N2)	19-Feb-2007	Germany	MK362160	MK362161	MK362162	MK362163	MK362164	MK362165	MK362166	MK362167
A/swine/Heiden/5945/2007 (H3N2)	22-Feb-2007	Germany	MK362168	MK362169	MK362170	MK362171	MK362172	MK362173	MK362174	MK362175
A/swine/Bondelum/IDT5959/2007 (H3N2)	26-Feb-2007	Germany	MK362176	MK362177	MK362178	GQ161151	MK362179	GQ161152	MK362180	MK362181
A/swine/Rinkerode/5967/2007 (H3N2)	02-Mar-2007	Germany	MK362182	MK362183	MK362184	MK362185	MK362186	MK362187	MK362188	MK362189
A/swine/Vreden/5982/2007 (H3N2)	08-Mar-2007	Germany	MK362190	MK362191	MK362192	MK362193	MK362194	MK362195	MK362196	MK362197
A/swine/Melle/6137/2007 (H3N2)	08-May-2007	Germany	MK362198	MK362199	MK362200	MK362201	MK362202	MK362203	MK362204	MK362205
A/swine/Friedberg/6140/2007 (H3N2)	11-May-2007	Germany	MK362206	MK362207	MK362208	MK362209	MK362210	MK362211	MK362212	MK362213
A/swine/Vreden/6235/2007 (H3N2)	14-Jun-2007	Germany	MK362214	MK362215	MK362216	MK362217	MK362218	MK362219	MK362220	MK362221
A/swine/Neuenrade/6259/2007 (H3N2)	28-Jun-2007	Germany	MK362222	MK362223	MK362224	MK362225	MK362226	MK362227	MK362228	MK362229
A/swine/Holzwickede/6267/2007 (H3N2)	20-Aug-2007	Germany	MK362230	MK362231	MK362232	MK362233	MK362234	MK362235	MK362236	MK362237
A/swine/Georgsmarienhütte/6391/2007 (H3N2)	27-Aug-2007	Germany	MK362238	MK362239	MK362240	MK362241	MK362242	MK362243	MK362244	MK362245
A/swine/Dörentrup/6485/2007 (H3N2)	25-Sep-2007	Germany	MK362246	MK362247	MK362248	MK362249	MK362250	MK362251	MK362252	MK362253
A/swine/Feldbach/6605/2007 (H3N2)	28-Oct-2007	Austria	MK362254	MK362255	MK362256	MK362257	MK362258	MK362259	MK362260	MK362261
A/swine/Balge/6607/2007 (H3N2)	30-Oct-2007	Germany	MK362262	MK362263	MK362264	MK362265	MK362266	MK362267	MK362268	MK362269
A/swine/Warendorf/6639a/2007 (H3N2)	08-Nov-2007	Germany	MK362270	MK362271	MK362272	MK362273	MK362274	MK362275	MK362276	MK362277
A/swine/Spenge/6671a/2007 (H3N2)	26-Nov-2007	Germany	MK362278	MK362279	MK362280	MK362281	MK362282	MK362283	MK362284	MK362285
A/swine/Isterberg/6682/2007 (H3N2)	29-Nov-2007	Germany	MK362286	MK362287	MK362288	MK362289	MK382290	MK362291	MK362292	MK362293
A/swine/Warendorf/6727/2007 (H3N2)	14-Dec-2007	Germany	MK362294	MK362295	MK362296	MK362297	MK362298	MK362299	MK362300	MK362301
A/swine/Röhrse/6764/2008 (H3N2)	11-Jan-2008	Germany	MK362302	MK362303	MK362304	MK362305	MK362306	MK362307	MK362308	MK362309
A/swine/Ölsen/6809/2008 (H3N2)	28-Jan-2008	Germany	MK362310	MK362311	MK362312	MK362313	MK362314	MK362315	MK362316	MK362317
A/swine/Meschede/6850/2008 (H3N2)	12-Feb-2008	Germany	MK362318	MK362319	MK362320	MK362321	MK362322	MK362323	MK362324	MK362325
A/swine/Bielefeld/6851/2008 (H3N2)	11-Feb-2008	Germany	MK362326	MK362327	MK362328	MK362329	MK362330	MK362331	MK362332	MK362333
A/swine/Brakel/6890/2008 (H3N2)	21-Feb-2008	Germany	MK362334	MK362335	MK362336	MK362337	MK362338	MK362339	MK362340	MK362341
A/swine/Reken/6891-4/2008 (H3N2)	12-Feb-2008	Germany	MK362342	MK362343	MK362344	MK362345	MK362346	MK362347	MK362348	MK362349
A/swine/Reken/6891-5/2008 (H3N2)	12-Feb-2008	Germany	MK362350	MK362351	MK362352	MK362353	MK362354	MK362355	MK362356	MK362357
A/swine/Nordkirchen/6904/2008 (H3N2)	22-Feb-2008	Germany	MK362358	MK362359	MK362360	MK362361	MK362362	MK362363	MK362364	MK362365
A/swine/Nienburg/6906/2008 (H3N2)	25-Feb-2008	Germany	MK362366	MK362367	MK362368	MK362369	MK362370	MK362371	MK362372	MK362373
A/swine/Ostbevern/6907/2008 (H3N2)	22-Feb-2008	Germany	MK362374	MK362375	MK362376	MK362377	MK362378	MK362379	MK362380	MK362381
A/swine/Melle/6908/2008 (H3N2)	25-Feb-2008	Germany	MK362382	MK362383	MK362384	MK362385	MK362386	MK362387	MK362388	MK362389
A/swine/Marsberg/6947/2008 (H3N2)	12-Mar-2008	Germany	MK362390	MK362391	MK362392	MK362393	MK362394	MK362395	MK362396	MK362397
A/swine/Büren/6971-1/2008 (H3N2)	29-Mar-2008	Germany	MK362398	MK362399	MK362400	MK362401	MK362402	MK362403	MK362404	MK362405
A/swine/Büren/6971-2/2008 (H3N2)	29-Mar-2008	Germany	MK362406	MK362407	MK362408	MK362409	MK362410	MK362411	MK362412	MK362413
A/swine/Langen/7114/2008 (H3N2)	26-May-2008	Germany	MK362414	MK362415	MK362416	MK362417	MK362418	MK362419	MK362420	MK362421
A/swine/Vreden/7123/2008 (H3N2)	28-May-2008	Germany	MK362422	MK362423	MK362424	MK362425	MK362426	MK362427	MK362428	MK362429
A/swine/Langen/7124/2008 (H3N2)	29-May-2008	Germany	MK362430	MK362431	MK362432	MK362433	MK362434	MK362435	MK362436	MK362437
A/swine/Rüthen/7174/2008 (H3N2)	14-Jun-2008	Germany	MK362438	MK362439	MK362440	MK362441	MK362442	MK362443	MK362444	MK362445
A/swine/Vreden/7175-1/2008 (H3N2)	12-Jun-2008	Germany	MK362446	MK362447	MK362448	MK362449	MK362450	MK362451	MK362452	MK362453
A/swine/Bad Griesbach/7186/2008 (H3N2)	13-Jun-2008	Germany	MK362454	MK362455	MK362456	MK362457	MK362458	MK362459	MK362460	MK362461
A/swine/Bad Wünnenberg/7188/2008 (H3N2)	19-Jun-2008	Germany	MK362462	MK362463	MK362464	MK362465	MK362466	MK362467	MK362468	MK362469
A/swine/Freren/7424/2008 (H3N2)	21-Aug-2008	Germany	MK362470	MK362471	MK362472	MK362473	MK362474	MK362475	MK362476	MK362477
A/swine/Paderborn/7588/2008 (H3N2)	24-Sep-2008	Germany	MK362478	MK362479	MK362480	MK362481	MK362482	MK362483	MK362484	MK362485
A/swine/Vreden/7613-1/2008 (H3N2)	30-Sep-2008	Germany	MK362486	MK362487	MK362488	MK362489	MK362490	MK362491	MK362492	MK362493
A/swine/Vreden/7613-2/2008 (H3N2)	30-Sep-2008	Germany	MK362494	MK362495	MK362496	MK362497	MK362498	MK362499	MK362500	MK362501
A/swine/Drensteinfurt/7825/2008 (H3N2)	27-Oct-2008	Germany	MK362502	MK362503	MK362504	MK362505	MK362506	MK362507	MK362508	MK362509
A/swine/Wiefelstede/7905/2008 (H3N2)	03-Nov-2008	Germany	MK362510	MK362511	MK362512	MK362513	MK362514	MK362515	MK362516	MK362517
A/swine/Rheine/7912a/2008 (H3N2)	04-Nov-2008	Germany	MK362518	MK362519	MK362520	MK362521	MK362522	MK362523	MK362524	MK362525
A/swine/Sassenberg/7957/2008 (H3N2)	10-Nov-2008	Germany	MK362526	MK362527	MK362528	MK362529	MK362530	MK362531	MK362532	MK362533
A/swine/Ostenfelde/8082/2008 (H3N2)	24-Nov-2008	Germany	MK362534	MK362535	MK362536	MK362537	MK362538	MK362539	MK362540	MK362541
A/swine/Borgentreich/8083/2008 (H3N2)	24-Nov-2008	Germany	MK362542	MK362543	MK362544	MK362545	MK362546	MK362547	MK362548	MK362549
A/swine/Iserlohn/8153/2008 (H3N2)	26-Nov-2008	Germany	MK362550	MK362551	MK362552	MK362553	MK362554	MK362555	MK362556	MK362557
A/swine/Damme/8154/2008 (H3N2)	01-Dec-2008	Germany	MK362558	MK362559	MK362560	MK362561	MK362562	MK362563	MK362564	MK362565
A/swine/Goldenstedt/8310a/2008 (H3N2)	11-Dec-2008	Germany	MK362566	MK362567	MK362568	MK362569	MK362570	MK362571	MK362572	MK362573
A/swine/Sassenberg/8395-1/2008 (H3N2)	29-Dec-2008	Germany	MK362574	MK362575	MK362576	MK362577	MK372578	MK362579	MK362508	MK362581
A/swine/Stemwede/8583/2009(H3N2)	19-Jan-2009	Germany	MK362582	MK362583	MK382584	MK362585	MK362586	MK362587	MK362588	MK362589
A/swine/Spenge/8584/2009 (H3N2)	17-Jan-2009	Germany	MK362590	MK362591	MK362592	MK362593	MK362594	MK362595	MK362596	MK362597
A/swine/Enger/8648/2009 (H3N2)	27-Jan-2009	Germany	MK362598	MK362599	MK362600	MK362601	MK362602	MK362603	MK362604	MK362605
A/swine/Warstein/9037/2009 (H3N2)	26-Feb-2009	Germany	MK362606	MK362607	MK362608	MK362609	MK362610	MK362611	MK362612	MK362613
A/swine/Garrel/9093/2009 (H3N2)	04-Mar-2009	Germany	MK362614	MK362615	MK362616	MK362617	MK362618	MK362619	MK362620	MK362621
A/swine/Willebadessen/9666/2009 (H3N2)	25-May-2009	Germany	MK362622	MK362623	MK362624	MK362625	MK362626	MK362627	MK362628	MK362629
A/swine/Deinstedt/9695/2009 (H3N2)	28-May-2009	Germany	MK362630	MK362631	MK362632	MK362633	MK362634	MK362635	MK362636	MK362637
A/swine/Tetenbüll/9718/2009 (H3N2)	05-Jun-2009	Germany	MK362638	MK362639	MK362640	MK362641	MK362642	MK362643	MK362644	MK362645
A/swine/Lohne/9719a/2009 (H3N2)	04-Jun-2009	Germany	MK362646	MK362647	MK362648	MK362649	MK362650	MK362651	MK362652	MK362653
A/swine/Kevelaer/9939/2009 (H3N2)	18-Jul-2009	Germany	MK362654	MK362655	MK363656	MK362657	MK362658	MK362659	MK362660	MK362661
A/swine/Telgte/10014/2009 (H3N2)	30-Jul-2009	Germany	MK362662	MK362663	MK362664	MK362665	MK362666	MK362667	MK362668	MK362669
A/swine/Westerstede/10029/2009 (H3N2)	03-Aug-2009	Germany	MK362670	MK362671	MK362672	MK362673	MK362674	MK362675	MK362676	MK362677
A/swine/Bottrop/10478a/2009 (H3N2)	21-Sep-2009	Germany	MK362678	MK362679	MK362680	MK362681	MK362682	MK362683	MK362684	MK362685
A/swine/Warendorf/10669/2009 (H3N2)	16-Oct-2009	Germany	MK362686	MK362687	MK362688	MK362689	MK362690	MK362691	MK362692	MK362693

A/swine/Warendorf/10670/2009(H3N2)	16-Oct-2009	Germany	MK362694	MK362695	MK362696	MK362697	MK362698	MK362699	MK362700	MK362701
A/swine/Sendenhorst/10745/2009 (H3N2)	13-Oct-2009	Germany	MK362702	MK362703	MK362704	MK362705	MK362706	MK362707	MK362708	MK362709
A/swine/Bösel/11289/2010 (H3N2)	15-Jan-2010	Germany	MK362710	MK362711	MK362712	MK362713	MK362714	MK362715	MK362716	MK362717
A/swine/Borken/11902/2010 (H3N2)	28-Apr-2010	Germany	MK362718	MK362719	MK362720	MK362721	MK362722	MK362723	MK362724	MK362725
A/swine/Friesoythe/12219/2010 (H3N2)	28-Jul-2010	Germany	MK362726	MK362727	MK362728	MK362729	MK362730	MK362731	MK362732	MK362733
A/swine/Schöppingen/12424/2010 (H3N2)	14-Sep-2010	Germany	MK362734	MK362735	MK362736	MK362737	MK362738	MK362739	MK362740	MK362741
A/swine/Lohne/12460/2010 (H3N2)	23-Sep-2010	Germany	MK362742	MK362743	MK362744	MK362745	MK362746	MK362747	MK362748	MK362749
A/swine/Georgsdorf/12866/2011 (H3N2)	03-Jan-2011	Germany	MK362750	MK362751	MK362752	MK362753	MK362754	MK362755	MK362756	MK362757
A/swine/Ascheberg/13209/2011 (H3N2)	28-Mar-2011	Germany	MK362758	MK362759	MK362760	MK362761	MK362762	MK362763	MK362764	MK362765
A/swine/Lohne/13611/2011 (H3N2)	09-Jul-2011	Germany	MK362766	MK362767	MK362768	MK362769	MK362770	MK362771	MK362772	MK362773
A/swine/Hörstel/13612-1/2011 (H3N2)	13-Jul-2011	Germany	MK362774	MK362775	MK362776	MK362777	MK362778	MK362779	MK362780	MK362781
A/swine/Hörstel/13612-2/2011 (H3N2)	13-Jul-2011	Germany	MK362782	MK362783	MK362784	MK362785	MK362786	MK362787	MK362788	MK362789
A/swine/Haren/13906/2011 (H3N2)	12-Sep-2011	Germany	MK362790	MK362791	MK362792	MK362793	MK362794	MK362795	MK362796	MK362797
A/swine/Billerbeck/14098/2011 (H3N2)	19-Oct-2011	Germany	MK362798	MK362799	MK362800	MK362801	MK362802	MK362803	MK362804	MK362805
A/swine/Bösel/14133/2011 (H3N2)	01-Nov-2011	Germany	MK362806	MK362807	MK362808	MK362809	MK362810	MK362811	MK362812	MK362813
A/swine/Atteln/14453/2011 (H3N2)	20-Dec-2011	Germany	MK362814	MK362815	MK362816	MK362817	MK362818	MK362819	MK362820	MK362821
A/swine/Haltern/14710/2012 (H3N2)	01-Feb-2012	Germany	MK362822	MK362823	MK362824	MK362825	MK362826	MK362827	MK362828	MK362829
A/swine/Steinfurt/14736/2012 (H3N2)	02-Feb-2012	Germany	MK362830	MK362831	MK362832	MK362833	MK362834	MK362835	MK362836	MK362837
A/swine/Coesfeld/14741-1/2012 (H3N2)	10-Feb-2012	Germany	MK362838	MK362839	MK362840	MK362841	MK362842	MK362843	MK362844	MK362845
A/swine/Sprakensehl/14752/2012 (H3N2)	09-Feb-2012	Germany	MK362846	MK362847	MK362848	MK362849	MK362850	MK362851	MK362852	MK362853
A/swine/Emsbüren/14771/2012 (H3N2)	09-Feb-2012	Germany	MK362854	MK362855	MK362856	MK362857	MK362858	MK362859	MK362860	MK362861
A/swine/Rüthen/14808/2012 (H3N2)	13-Feb-2012	Germany	MK362862	MK362863	MK362864	MK362865	MK362866	MK362867	MK362868	MK362869
A/swine/Bad Wünnenberg/14810/2012 (H3N2)	19-Feb-2012	Germany	MK362870	MK362871	MK362872	MK362873	MK362874	MK362875	MK362876	MK362877
A/swine/Bad Wünnenberg/14829/2012 (H3N2)	22-Feb-2012	Germany	MK362878	MK362879	MK362880	MK362881	MK362882	MK362883	MK362884	MK362885
A/swine/Bösingen/16789/2012 (H3N2)	14-Dec-2012	Germany	MK362886	MK362887	MK362888	MK362889	MK362890	MK362891	MK362892	MK362893
A/swine/Salzkotten/17022/2013 (H3N2)	18-Jan-2013	Germany	MK362894	MK362895	MK362896	MK362897	MK362898	MK362899	MK362900	MK362901
A/swine/Parschalling/17042/2013 (H3N2)	19-Jan-2013	Germany	MK362902	MK362903	MK362904	MK362905	MK362906	MK362907	MK362908	MK362909
A/swine/Lohne/17104/2013 (H3N2)	29-Jan-2013	Germany	MK362910	MK362911	MK362912	MK362913	MK362914	MK362915	MK362916	MK362917
A/swine/Steinfurt/17118/2013 (H3N2)	05-Feb-2013	Germany	MK362918	MK362919	MK362920	MK362921	MK362922	MK362923	MK362924	MK362925
A/swine/Nottuln/17261/2013 (H3N2)	21-Feb-2013	Germany	MK362926	MK362927	MK362928	MK362929	MK362930	MK362931	MK362932	MK362933
A/swine/Melle/17290/2013 (H3N2)	26-Feb-2013	Germany	MK362934	MK362935	MK362936	MK362937	MK362938	MK362939	MK362940	MK362941
A/swine/Bad Wünnenberg/17298/2013 (H3N2)	12-Feb-2013	Germany	MK362942	MK362943	MK362944	MK362945	MK362946	MK362947	MK362948	MK362949
A/swine/Boitzenborstel/17524/2013 (H3N2)	25-Mar-2013	Germany	MK362950	MK362951	MK362952	MK362953	MK362954	MK362955	MK362956	MK362957
A/swine/Telgte/17626/2013 (H3N2)	11-Apr-2013	Germany	MK362958	MK362958	MK362959	MK362960	MK362961	MK362963	MK362964	MK362965
A/swine/Bunnik/17685/2013 (H3N2)	15-Apr-2013	Netherlands	MK362966	MK362967	MK362968	MK362969	MK362970	MK362971	MK362972	MK362973
A/swine/Rhede/17728/2013 (H3N2)	22-Apr-2013	Germany	MK362974	MK362975	MK362976	MK362977	MK362978	MK362979	MK362980	MK362981
A/swine/Borchen/17851/2013 (H3N2)	29-Apr-2013	Germany	MK362982	MK362983	MK362984	MK362985	MK362986	MK362987	MK362988	MK362989
A/swine/Märschendorf/17986/2013 (H3N2)	04-Jun-2013	Germany	MK362990	MK362991	MK362992	MK362993	MK362994	MK362995	MK362996	MK362997
A/swine/Nordwalde/18011/2013 (H3N2)	11-Jun-2013	Germany	MK362998	MK362999	MK363000	MK363001	MK363002	MK363003	MK363004	MK363005
A/swine/Nottuln/18090/2013 (H3N2)	25-Jun-2013	Germany	MK363006	MK363007	MK363008	MK363009	MK363010	MK363011	MK363012	MK363013
A/swine/Molbergen/18109/2013 (H3N2)	21-Jun-2013	Germany	MK363014	MK363015	MK363016	MK363017	MK363018	MK363019	MK363020	MK363021
A/swine/Nordwalde/18134/2013 (H3N2)	27-Jun-2013	Germany	MK363022	MK363023	MK363024	MK363025	MK363026	MK363027	MK363028	MK363029
A/swine/Altenberge/18142/2013 (H3N2)	03-Jul-2013	Germany	MK363030	MK363031	MK363032	MK363033	MK363034	MK363035	MK363036	MK363037
A/swine/Glandorf/18169/2013 (H3N2)	03-Jul-2013	Germany	MK363038	MK363039	MK363040	MK363041	MK363042	MK363043	MK363044	MK363045
A/swine/Nieheim/18259/2013 (H3N2)	16-Jul-2013	Germany	MK363046	MK363047	MK363048	MK363049	MK363050	MK363051	MK363052	MK363053
A/swine/Nordwalde/18367/2013 (H3N2)	02-Aug-2013	Germany	MK363054	MK363055	MK363056	MK363057	MK363058	MK363059	MK363060	MK363061
A/swine/Stapelfeld/18554/2013 (H3N2)	04-Sep-2013	Germany	MK363062	MK363063	MK363064	MK363065	MK363066	MK363067	MK363068	MK363069
A/swine/Nordwalde/18569/2013 (H3N2)	04-Sep-2013	Germany	MK363070	MK363071	MK363072	MK363073	MK363074	MK363075	MK373076	MK363077
A/swine/Roswinkel/18812/2013 (H3N2)	01-Oct-2013	Netherlands	MK363078	MK363079	MK363080	MK363081	MK363082	MK363083	MK363084	MK363085
A/swine/Warendorf/18914/2013 (H3N2)	16-Oct-2013	Germany	MK363086	MK363087	MK363088	MK363089	MK363090	MK363091	MK363092	MK363093
A/swine/Rietberg/19732/2014 (H3N2)	19-Feb-2014	Germany	MK363094	MK363095	MK363096	MK363097	MK363098	MK363099	MK363100	MK363101
A/swine/Telgte/19839/2014 (H3N2)	11-Mar-2014	Germany	MK363102	MK363103	MK363104	MK363105	MK363106	MK363107	MK363108	MK363109
A/swine/Telgte/19851/2014 (H3N2)	13-Mar-2014	Germany	MK363110	MK363111	MK363112	MK363113	MK363114	MK363115	MK363116	MK363117
A/swine/Herzlake/19959/2014 (H3N2)	02-Apr-2014	Germany	MK363118	MK363119	MK363120	MK363121	MK363122	MK363123	MK363124	MK363125
A/swine/Telgte/19994/2014 (H3N2)	15-Apr-2014	Germany	MK363126	MK363127	MK363128	MK363129	MK363130	MK363131	MK363132	MK363133
A/swine/Zweiflingen/20028/2014 (H3N2)	23-Apr-2014	Germany	MK363134	MK363135	MK363136	MK363137	MK363138	MK363139	MK363140	MK363141
A/swine/Rhede/20749/2014 (H3N2)	13-Oct-2014	Germany	MK363142	MK363143	MK363144	MK363145	MK363146	MK363147	MK363148	MK363149
A/swine/Goldenstedt/20754/2014 (H3N2)	16-Oct-2014	Germany	MK363150	MK363151	MK363152	MK363153	MK363154	MK363155	MK363156	MK363157
A/swine/Zevenaar/21030/2014 (H3N2)	26-Nov-2014	Netherlands	MK363158	MK363159	MK363160	MK363161	MK363162	MK363163	MK363164	MK363165
A/swine/Borken/21121/2015 (H3N2)	07-Jan-2015	Germany	MK363166	MK363167	MK363168	MK363169	MK363170	MK363171	MK363172	MK363173
A/swine/Günne/21206/2015 (H3N2)	21-Jan-2015	Germany	MK363174	MK363175	MK363176	MK363177	MK363178	MK363179	MK363180	MK363181
A/swine/Borken/21229/2015 (H3N2)	22-Jan-2015	Germany	MK363182	MK363183	MK363184	MK363185	MK363186	MK363187	MK363188	MK363189
A/swine/Wielen/21300/2015 (H3N2)	12-Feb-2015	Germany	MK363190	MK363191	MK363192	MK363193	MK363194	MK363195	MK363196	MK363197
A/swine/Spain/21350/2015 (H3N2)	25-Feb-2015	Spain	MK363198	MK363199	MK363200	MK363201	MK363202	MK363203	MK363204	MK363205
A/swine/Heiden/21511/2015 (H3N2)	30-Mar-2015	Germany	MK363206	MK363207	MK363208	MK363209	MK363210	MK363211	MK363212	MK363213
A/swine/Friesoythe/21674/2015 (H3N2)	11-May-2015	Germany	MK363214	MK363215	MK363216	MK363217	MK363218	MK363219	MK363220	MK363221
A/swine/Friesoythe/21704/2015 (H3N2)	18-May-2015	Germany	MK363222	MK363223	MK363224	MK363225	MK363226	MK363227	MK363228	MK363229
A/swine/Dreierwalde/21715/2015 (H3N2)	13-May-2015	Germany	MK363230	MK363231	MK363232	MK363233	MK363234	MK363235	MK363236	MK363237
A/swine/Rietberg/22412/2015 (H3N2)	16-Nov-2015	Germany	MK363238	MK363239	MK363240	MK363241	MK363242	MK363243	MK363244	MK363245
A/swine/Cloppenburg/22418/2015 (H3N2)	17-Nov-2015	Germany	MK363246	MK363247	MK363248	MK363249	MK363250	MK363251	MK363252	MK363253
A/swine/Hassendorf/2929/2004 (H3N1)	26-Jan-2004	Germany	MK332395	MK332396	MK332397	MK332398	MK332399	MK332400	MK332401	MK332402
A/swine/Lünne/4529/2005 (H3N1)	06-Oct-2005	Germany	MK332403	MK332404	MK332405	MK332406	MK332407	MK332408	MK332409	MK332410
A/swine/Feldbach/4589/2005 (H3N1)	20-Oct-2005	Austria	MK332411	MK332412	MK332413	MK332414	MK332415	MK332416	MK332417	MK332418
A/swine/Holtrup/6358/2007 (H3N1)	09-Aug-2007	Germany	MK332419	MK332420	MK332421	MK332422	MK332423	MK332424	MK332425	MK332426
A/swine/Stresow/7919a/2008 (H3N1)	03-Nov-2008	Germany	MK332427	MK332428	MK332429	MK332430	MK332431	MK332432	MK332433	MK332434
A/swine/Würusreuth/IDT10990/2009 (H3N1)	17-Nov-2009	Germany	KC222513	KC222514	KC222515	KC222516	KC222517	KC222518	KC222519	KC222520
